# Supplementary material for: Randomised feasibility trial into the effects of low-frequency electrical muscle stimulation in advanced heart failure patients
Source: BMJ Open. 2017 Aug 11;7(8):e016148. doi: 10.1136/bmjopen-2017-016148 (PMC5629639; doi:10.1136/bmjopen-2017-016148)
Supplement: Author's manuscript [file bmjopen-2017-016148.draft_revisions.pdf]

BMJ Open

BMJ Open is committed to open peer review. As part of this commitment we make the peer review history of every article we publish publicly available.

When an article is published we post the peer reviewers' comments and the authors' responses online. We also post the versions of the paper that were used during peer review. These are the versions that the peer review comments apply to.

The versions of the paper that follow are the versions that were submitted during the peer review process. They are not the versions of record or the final published versions. They should not be cited or distributed as the published version of this manuscript.

BMJ Open is an open access journal and the full, final, typeset and author-corrected version of record of the manuscript is available on our site with no access controls, subscription charges or pay-per-view fees (http://bmjopen.bmj.com).

If you have any questions on BMJ Open's open peer review process please email editorial.bmjopen@bmj.com

BMJ Open

Randomised pilot study into the effects of low frequency electrical muscle stimulation in advanced heart failure patients

Journal:	*BMJ Open*
Manuscript ID	bmjopen-2017-016148
Article Type:	Research
Date Submitted by the Author:	26-Jan-2017
Complete List of Authors:	Ennis, Stuart; University Hospitals Coventry and Warwickshire NHS Trust, Cardiac Rehabilitation; Cardiff Metropolitan University, Cardiff Centre for Exercise & Health<br>McGregor, Gordon; University Hospitals Coventry and Warwickshire NHS Trust, Cardiac Rehabilitation; Coventry University Faculty of Health and Life Sciences<br>Hamborg, Thomas; University of Warwick Warwick Medical School,<br>Jones, Helen; Liverpool John Moores University, sport and exercise sciences<br>Shave, Rob; Cardiff Metropolitan University, Physiology and Health<br>Singh, Sally; University Hospitals of Leicester NHS Trust, Cardiac/Pulmonary Rehabilitation<br>Banerjee, Prithwish; University Hospitals Coventry and Warwickshire NHS Trust, Department of Cardiology; Coventry University
**Primary Subject Heading**:	Sports and exercise medicine
Secondary Subject Heading:	Cardiovascular medicine
Keywords:	neuromuscular electrical stimulation, cardiac rehabilitation, functional electrical stimulation, chronic heart failure, electrical muscle stimulation

Note: The following files were submitted by the author for peer review, but cannot be converted to PDF. You must view these files (e.g. movies) online.

MAGIC PANTS Protocol V3 30 JUN 2014BMJ open.docm

SCHOLARONE™ Manuscripts

Randomised pilot study into the effects of low frequency electrical muscle stimulation in advanced heart failure patients

Stuart Ennis[1], Gordon McGregor[1,6], Thomas Hamborg [2], Helen Jones[3], Robert Shave[4], Sally J Singh[5,6] and Prithwish Banerjee[1,6].

For peer review only

Abstract .

Background: Chronic heart failure patients have limited treatment options and struggle to adhere to exercise training programmes. Low Frequency Electrical Muscle Stimulation (LF-EMS) may have the potential to reduce breathlessness and increase exercise capacity in this cohort. However, the practical feasibility of a randomised controlled trial of LF-EMS in this debilitated population has not been established.

Methods: Using a double blind, randomised study design, 60 severe heart failure patients (New York Heart Association class III-IV) were assigned to 8 weeks (5 x 60 mins per week) of either LF-EMS intervention (4Hz, continuous) or SHAM placebo (skin level stimulation only) of the quadriceps and hamstrings muscles. Participants used the LF-EMS straps at home and were supervised weekly. Recruitment, adherence and tolerability to the intervention were measured during the trial as well as physiological outcomes (6 minute walk, quadriceps strength, quality of life and physical activity).

Results: 12 (20%) of the 60 patients (4 LF-EMS, 8 SHAM) withdrew. Reasons for dropout were: deterioration in health (n= 6) family problems (n=2) device implantation (n=1) and found the stimulation intolerable (n=3) Forty One patients (68.3 %), adhered to the protocol for at least 70% of the sessions**.** The physiological measures indicated improvement in the LF-EMS group in 6 minute walk distance, and quality of life, although not significantly.

Conclusion: Severe heart failure patients can be recruited to and adhere to LF-EMS studies and find it tolerable. A larger Randomised Controlled Trial (RCT) in the advanced heart failure population is therefore feasible. The trial results showed non-significant improvement of EMS in 6 minute walk distance, and quality of life, both of which could be considered as primary outcomes in a larger trial.

Trial registration number: ISRCTN16749049

Strengths and Limitations

1. To our Knowledge, this was the first study to evaluate the design of a study into LF-EMS in advanced (NYHA III-IV) heart failure patients
2. Analysis of recruitment, retention and adherence in this hard to reach group contributes useful knowledge to the heart failure literature on how practical exercise interventions could be implemented.
3. Participants were instructed to adhere to the 'maximum tolerable intensity' during LF-EMS sessions. Due to considerable individual differences in the subjective perception of discomfort associated with EMS, It is therefore likely that there was variability in the intensity that individuals received
4. Because this study focused on the feasibility of using LF-EMS in advanced heart failure patients for 8 weeks, the long term sustainability of this intervention is unclear.

For peer review only - http://bmjopen.bmj.com/site/about/guidelines.xhtml

Introduction

Chronic Heart Failure (CHF) affects approximately 26 million people worldwide, [1] and is associated with a poor prognosis; 30- 40% of patients diagnosed with heart failure die within a year. [2] Patients in New York Heart Association (NYHA) class III/IV are unable to perform the simplest daily activities, become depressed and have a poor quality of life.[3]

Regular aerobic exercise reduces breathlessness and muscle dysfunction for individuals with CHF whilst improving exercise capacity.[4,5,6] According to the ExTraMATCH meta-analysis,[7] exercise training leads to a 35% relative reduction in mortality, similar to the effects of beta-blockers[8] and ACE inhibitors.[9] However, those with advanced CHF are often so limited that they are unable to gain the holistic benefits of exercise.[4,7]

Electrical Muscle Stimulation (EMS) may provide an alternative rehabilitative therapy for this group. In patients with mild to moderate CHF, EMS can improve muscle strength of the legs, exercise capacity and quality of life. [10,11,12] Low frequency (4-5Hz) electrical muscle stimulation (LF-EMS) produces shivering-like sub-tetanic muscle contractions that can stimulate an aerobic response equivalent to 51% of maximal oxygen uptake.[13] Therapeutic levels of aerobic exercise can thus be achieved passively by LF-EMS,[14] and it has been shown to be comfortable and well tolerated in healthy individuals and those with mild to moderate CHF. [15,16] However, the impact of LF-EMS in advanced heart failure (NYHA class III/IV) patients is currently unknown. As advanced heart failure patients have shown poor uptake and adherence to intervention studies,[17] a preliminary study was needed to determine the feasibility of LF-EMS in this patient cohort prior to the development of a large-scale definitive trial.

Based upon recommendations for good practice in the design of pilot and feasibility studies [18] this study was undertaken with the following aims: To (a) test the robustness of the study protocol for a potential future trial, (b) estimate rates of recruitment, consent and retention, (c) determine the tolerability of the LF-EMS intervention and the effectiveness of the sham placebo in the NYHA III/IV CHF population, and (d) gain initial estimates of the efficacy of LF-EMS for all potential primary outcomes. This can be used for sample size calculations in future substantive trials.

For peer review only

Methods

Experimental Design

This feasibility study used a double blind parallel group randomised control design. Participants were randomised to either LF-EMS or 'sham' placebo for a period of eight weeks and blinded to group allocation. Outcomes were assessed at baseline (pre randomisation), eight weeks and 20 weeks follow-up.

Recruitment and screening

Between October 2013 and March 2015, University Hospital Coventry and Warwickshire, (UHCW) Hospital NHS Trust heart failure clinics lists were screened for patients fulfilling the eligibility criteria for the study. Sixty eligible participants were recruited. The study conformed to the Declaration of Helsinki and was approved by the local NHS Ethics Committee. All participants provided written informed consent.

Randomisation

The trial statistician, in conjunction with Warwick Clinical Trials Unit generated the randomisation sequence remotely (by computer) using permuted block randomisation. Group allocation was concealed from outcomes assessors and participants.

Participants

Male and female adults, >18 years old, with stable CHF, documented by echocardiography of left ventricular systolic dysfunction (ejection fraction < 40%) were eligible for the study. All participants had New York Heart Association (NYHA) functional class III-IV symptoms. Participants were required to be medically stable, defined as the absence of hospital admission or alterations in medical therapy within the preceding two weeks. Exclusion criteria for safety and practical reasons were: (1) presence of implantable cardiac devices, (2) serious cardiac arrhythmias,(3) neurological disorders or previous stroke significant enough to limit exercise, (4) orthopaedic problems that prevented walking, (5) neuromuscular disease, (6) dementia or (7) a mid-thigh circumference of more than 50cm (due to the size of the LF-EMS straps).

LF-EMS Stimulation

The LF-EMS equipment (Biomedical Research Limited, Galway, Ireland) consisted of a pair of neoprene straps containing built-in adhesive gel electrodes. The equipment is CE marked under the European Medical Device Directive. The stimulator current waveform was designed to produce rhythmical contractions in the leg muscle groups occurring at a pulse frequency of 4-5Hz (pulse width: 620μs). The maximum peak output pulse current used was 140mA.

LF-EMS intervention

Participants used the LF-EMS or sham placebo for one hour, five times a week, for eight consecutive weeks. Of the five hourly sessions per week, four were

For peer review only - http://bmjopen.bmj.com/site/about/guidelines.xhtml

completed unsupervised in the participant's own home. The remaining session was conducted in a cardiac rehabilitation outpatient setting under the supervision of an exercise physiologist. The LF-EMS technology was retrospectively interrogated (i.e. at the weekly supervised sessions) to report date, frequency, duration and stimulation intensity.

'Sham' Placebo intervention

In the sham arm of the study, participants were provided with identical straps and electrodes. In contrast to the LF-EMS group the controller was programmed to deliver a very low level of stimulation (Frequency: 99Hz, pulse width: 150µs, maximum current amplitude: 7.3mA). This provided sensory input to the skin surface but little or no muscle activation. Participants in the sham group had the same induction, supervision and follow-up as the intervention arm.

Outcome Measures

Feasibility criteria

In relation to the design of pilot and feasibility studies, Thabane et al,[19] recommends stipulating criteria for success '*a priori*'. The feasibility criteria were:

1. *Recruitment rate* – At least 40% of eligible participants recruited to the trial

2. *Retention* – no more than 33% of participants drop out during the intervention period.

3. *Adherence* – 66% of participants tolerate the intervention and adhere to the protocol for ≥70% of the intervention period.

4. *Placebo efficacy*- Participants would be able to guess their group allocation no more often than would be expected by chance.

Secondary outcomes

Six Minute Walk Test (6MWT).

The 6MWT was conducted in accordance with the American Thoracic Society (ATS) guidelines.[20] Participants were instructed to walk as far as possible in six minutes along a 30m, flat, obstacle free corridor, turning 180 degrees at the end of every 30m. Standardised instructions and verbal encouragement were given.

Isometric muscle strength

A hand held dynamometer (MicroFET2 Torque/Force indicator, Hoggan Health Industries, Utah, US) validated for assessing functional leg strength in elderly populations was used.[21] Participants sat in an elevated chair and were instructed to maximally extend the knee while the assessor provided an equal and opposite

For peer review only

For peer review only - http://bmjopen.bmj.com/site/about/guidelines.xhtml

resistive force, against the lower shin. Mean force generated was measured in Newtons .

Quality of Life: Minnesota Living with Heart Failure Questionnaire (MLHFQ)

The MLWHF questionnaire is a disease validated questionnaire,[22] that has been extensively used in heart failure studies. Questionnaire scores range from 0 to 105, with higher scores reflecting lower Quality of life. Participants were asked to answer each question based on their perception of health in the week previous to testing.

Physical Activity levels

Physical activity levels were measured by the Bodymedia© SenseWear Pro3 Armband. The multi-plane accelerometer was worn continuously for the seven days prior to testing to determine Total Energy Expenditure (TEE) per 24hr period was used as the main indicator of physical activity.

Safety: Blood test

Venous blood samples were taken at baseline, four weeks and eight weeks to assess creatine kinase (CK), urea, and electrolytes. Participants would discontinue the trial if levels exceeded the upper limit of normal reference ranges

Data analysis

Data analyses for the feasibility objectives of this study were descriptive, based on the pre-determined levels specified above. Confidence intervals (set at 95%) were calculated for all secondary outcome measures in both groups and paired two-sample t-test conducted for between group comparisons. Intent-to-treat (ITT) analysis was employed in this study as is recommended for clinical trials.[24]

For peer review only

190 **Results**

191 **Feasibility criteria outcomes**

192 *Recruitment*

193 There were 171 eligible participants identified in the Coventry and
194 Warwickshire area from November 2013 - April 2015. Sixty of 171 eligible
195 participants (35.08%) were recruited to the trial. Participants were randomised and
196 started on the trial during this period and were followed up until data collection
197 finished in August 2015. Participant characteristics are presented in Table 1.

198

For peer review only

Table 1. Baseline demographic and clinical characteristics of the LF-EMS and sham placebo groups. Data presented as mean ± SD or absolute number and percent.

**Demographics**	**LF-EMS (n-30)**	**Sham (n=30)**
n Male	20 (66%)	22 (73%)
Age (yrs)	66.5 ± 7.8	66.8 ± 13.5
Body Mass Index (kg/m^2)	30.1 ± 4.9	27.8 ± 4.8
**Clinical**		
BP_{sys} (mmHg)	118 ± 16	126 ± 17
BP_{dia} (mmHg)	69 ± 9	74 ± 14
NYHA III	24 (80%)	22 (73%)
NYHA IV	6 (20%)	8 (26%)
**Comorbidities**		
Prev MI/PCI/CABG	17 (56%)	11 (36%)
Diabetes	12 (40%)	10 (33%)
COPD	9 (30%)	8 (26%)
AF	20 (66%)	16 (53%)
Hypertension	13 (43%)	10 (33%
CKD	5 (16%)	13 (43%)

BP_{sys} (mmHg), systolic blood pressure; BP_{dia} (mmHg), diastolic blood pressure; NYHA, New York Heart association; MI, myocardial infarction; PCI, percutaneous coronary intervention; CABG, coronary artery bypass graft surgery; COPD, chronic obstructive pulmonary disease; AF, atrial fibrillation; CKD, chronic kidney disease;

For peer review only

Retention

Twelve of the 60 participants (4 LF-EMS, 8 sham) (20%) withdrew and did not finish the intervention period (See Fig 1). Of these, only three found the intervention intolerable (1 LF-EMS, 2 sham). Other reasons for dropout were: deterioration in health (n= 6) family problems (n=2) and implantation of a cardioverter defibrillator (ICD) (n=1). Only 22 (45%) of those completing the intervention period returned for follow-up testing at 20 weeks. Reasons for non-follow-up were: deterioration in health (n=9), excluded due to implantation of cardiac resynchronisation therapy device (n=2), declined to take part without further explanation (n=13), and could not be contacted after repeated attempts (n= 3).

For peer review only

For peer review only - http://bmjopen.bmj.com/site/about/guidelines.xhtml

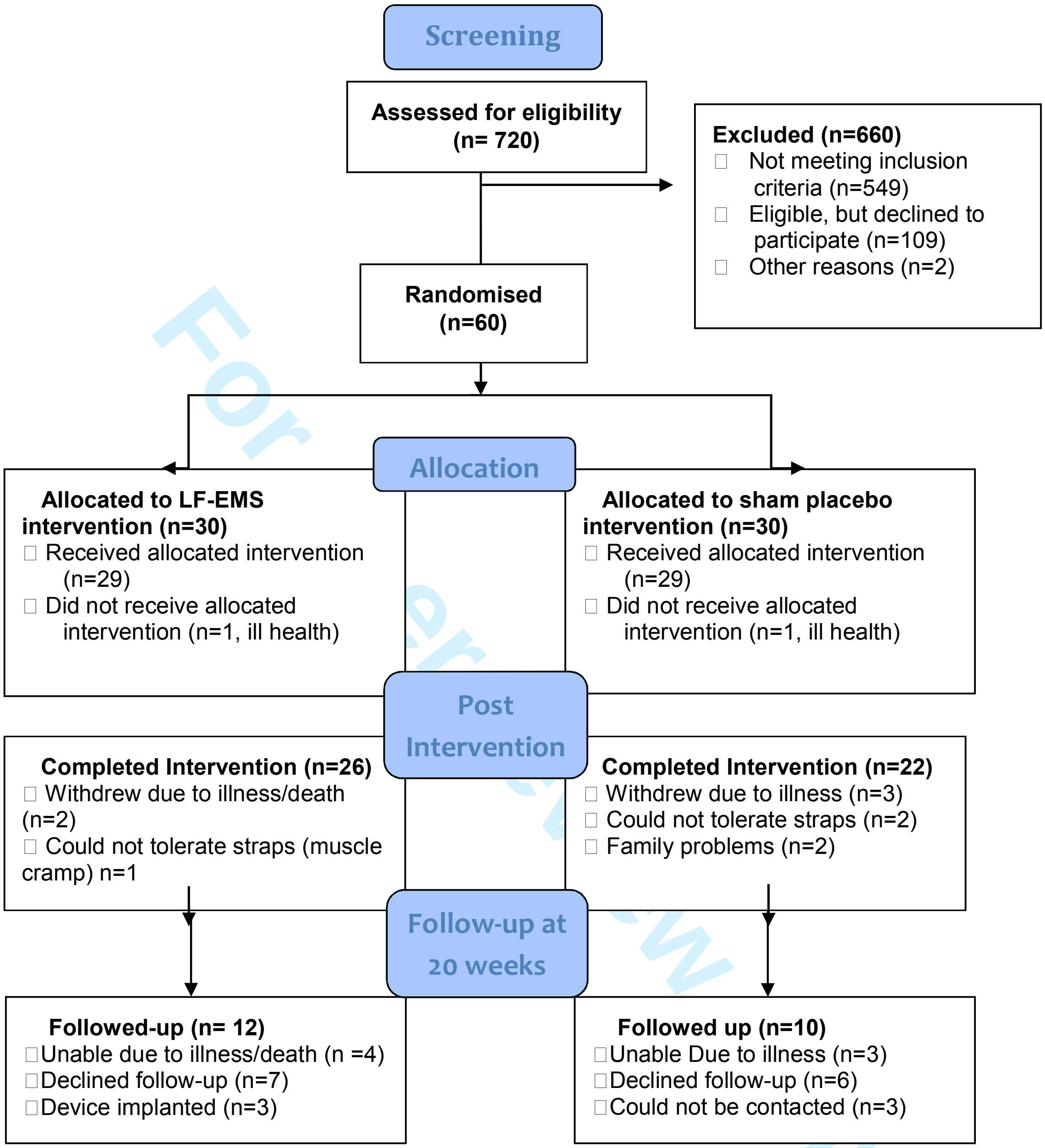

Figure 1. Flow diagram of a single centre blinded parallel group randomised feasibility trial of electrical muscle stimulation versus sham placebo in severe heart failure patients.

For peer review only - http://bmjopen.bmj.com/site/about/guidelines.xhtml

Adherence

Forty one (85.4 %) of the 48 participants (22-LF-EMS, 19-SHAM) who completed the intervention period (68.3% of the total sample) adhered to the strict protocol for the majority (>70%) of the eight weeks. Interrogation of the LF-EMS controllers revealed that participants in the LF-EMS group became more tolerant to the intervention; mean stimulation intensity increased from 57.79mA (95%CI: 51.16 to 64.42) during week 1 of the study to 84.86mA (95%CI: 75.44 to 94.28) by week 8, an improvement of 46.5%.

'Sham' Placebo

The sham placebo for the study appeared to be convincing as only 61% of participants guessed their treatment group correctly. The 95% confidence interval for the proportion of participants guessing correctly was (46% to 74%) and thus not significantly different from 50% which would be expected by chance. Furthermore, participants demonstrated an inclination to guess that they were randomised to LF-EMS regardless of group allocation.

Safety

No abnormalities were detected in CK, urea or electrolytes taken before, during or after the study. Likewise, no adverse events due to the intervention were recorded in either group.

Secondary Outcomes

Table 2 shows the mean values of outcome measures at each time point. There were no significant differences between groups in the change from baseline for any of the secondary outcome variables (Table 3). Non-significant improvements after LF-EMS treatment (8 week time point) were observed in 6 MWD and Quality of life.

For peer review only

Table 2: Outcome measurements – Time point averages and 95% confidence intervals (CI)

Outcome	Time point	LF-EMS	Sham
Mean 6 MWD (metres) [95% CI]	Baseline	283 [237 – 328]	290 [243 – 337]
	(n)	29	29
	8 weeks	312 [262 – 362]	318 [270 – 365]
	(n)	26	22
	20 weeks	257 [173 – 342]	226 [126 – 325]
	(n)	12	10
(Mean leg strength (newtons) [95% CI]	Baseline	234.3 [196.5 – 272.]	297.5 [253 – 342]
	(n)	29	29
	8 weeks	224.9 [187.5 – 262.3]	321 [267.8 – 374.3]
	(n)	25	22
	20 weeks	181.6 [131.7 – 231.5]	207.1 [148.6 – 265.7]
	(n)	11	10
Mean QoL (score) [95% CI]	Baseline	53.1 [42.7 – 63.5]	50 [40 – 60.1]
	(n)	28	29
	8 weeks	43.9 [34.2 – 53.5]	43.1 [30.9 – 55.3]
	(n)	25	22
	20 weeks	51.7 [31.6 – 71.8]	37.0 [16.9 – 57]
	(n)	12	10
Mean TEE (joules) *[95% CI]*	Baseline	63,438 [56,170 – 70,705]	65,371 [59675 – 71,067]
	(n)	25	27
	8 weeks	59,783 [51,094 – 68,471]	59,687 [50,630 – 68,745]
	(n)	19	17
	20 weeks	61,878 [53,345 – 70,410]	63,541 [55,795 – 71,287]
	(n)	7	6

6 MWD, 6 minute walk distance, QoL, quality of life; TEE, Total Energy Expenditure

For peer review only

For peer review only

For peer review only - http://bmjopen.bmj.com/site/about/guidelines.xhtml

Table 3: -Changes from baseline averages and 95% confidence intervals (CI)

Outcome	Time point	LF-EMS	Sham	p-value
Mean 6 MWD (metres) [95% CI]	Baseline to 8 weeks	24 [9 – 40]	9 [-4 – 22]	0.1366
	(n)	26	22	
	Baseline to 20 weeks	0 [-32 – 31]	-26.30 [-63 – 11]	0.2409
	(n)	12	10	
(Mean leg strength (newtons) [95% CI]	Baseline to 8 weeks	-9.2 [-28.9 – 10.5]	6.0 [-19.3 – 31.4]	0.3244
	(n)	25	22	
	Baseline to 20 weeks	-43.4 [-78.7 – -8.2]	-74.1 [-116.3 – -31.9]	0.2223
	(n)	11	10	
Mean QoL (score) [95% CI]	Baseline to 8 weeks	-7.6 [-15.5 – 0.3]	-4.7 [-10.5 – 1.0]	0.5505
	(n)	25	22	
	Baseline to 20 weeks	1.5 [-12.5 – 15.7]	-14.0 [-34 – 6]	0.1610
	(n)	12	10	
Mean TEE (joules) [95% CI]	Baseline to 8 weeks	-4635 [-3963 – 4692]	-8168 [-14,342 – -1995]	0.5108
	(n)	19	17	
	Baseline to 20 weeks	1686 [-6435 – 9809]	4177 [-7695 – 16,050]	0.6634
	(n)	7	6	

6 MWD, 6 minute walk distance; QoL, quality of life; TEE, Total Energy Expenditure

For peer review only

Discussion

The predetermined criteria for proceeding to a larger trial were achieved for dropout (20%), adherence (68.3%) and sham placebo efficacy (61.53% participants guessed correctly). However, only 35.06% of eligible patients were recruited, below the target of 40%. Initial outcome measures revealed no significant difference between intervention and placebo groups, although there was a non-significant improvement in 6MWD and quality of life after LF-EMS.

Feasibility outcomes

Recruitment

Percentage uptake (35.06%) of eligible patients in the study was below the predetermined criteria of 40%. This is similar to the poor uptake of conventional cardiac rehabilitation (CR) nationally in the UK: less than 40% of eligible post myocardial infarction patients and those undergoing elective percutaneous coronary interventions (PCI) accessed CR in the most recent National Audit of Cardiac Rehabilitation.[26]

Retention/adherence/tolerance

One strength of this study is the good level of adherence (68.3%) and retention (80%) compared with other clinical studies; In the HF-ACTION trial,[27] only 40% of patients in the exercise group (n=1159) reported adherence to recommended training volumes after three months. This may have been because of the ease of independent use at home of LF-EMS, in combination with the weekly supervised sessions with an exercise physiologist The patients recruited in the present trial were more debilitated yet they engaged more with LF-EMS than those in the HF-ACTION trial, [27] suggesting that LF-EMS maybe more acceptable to this population than conventional exercise.

Tolerance to the LF-EMS intervention improved during the study. Mean current intensity increased by 46% from week one to week eight. This tolerance effect is in keeping with an earlier study by Crognale, et al,[13] that showed a 20% increase in healthy active adults. The active adults tolerated higher absolute stimulation levels than in this study, both before and after habituation, suggesting that advanced CHF patients are subjectively less tolerant to LF-EMS than a healthy population. Vivodtzev and colleagues,[28] examined factors determining tolerance of EMS in pulmonary patients. The study reported that lower tolerance to EMS was associated with greater severity of condition, fat free mass and inflammatory response. It is possible that the same is true in the CHF population but more research is needed to confirm this.

Secondary outcomes

For peer review only - http://bmjopen.bmj.com/site/about/guidelines.xhtml

Quality of life (MLHFQ) improved in both groups after the intervention. This may, in part, relate to the psychosocial benefits of engaging with researchers regularly in the cardiac rehabilitation facility.[29] The placebo effect of both interventions and its influence on patients' perception of well-being should not be underestimated.

The non-significant improvements in exercise capacity as measured by 6 minute walk were smaller than those in a meta-analysis of EMS in heart failure patients by Smart, Dieberg and Giallulria.[10] These authors reported a combined improvement in 6MWD of 46.9m vs usual care or placebo, compared to the effect size of 13.2m in this study. However, patients in this study were more symptomatic than those included in the meta-analysis,[10] and thus had a lower baseline exercise capacity (286m vs 342m.) Nevertheless, the relative increase (5%) in walk distance of participants in the LF-EMS group is within the measurement error associated with this test,[30] and probably should not be considered clinically significant.[31] The extrapolation from these results that severe CHF patients are beyond help from EMS maybe premature; a longer training period maybe required to show meaningful changes in exercise capacity, particularly as some participants took longer to tolerate meaningful EMS intensities than others.

Based on previous research by Banerjee et al,[15,16] and numerous high frequency EMS studies,[32,33,12] improvement in leg strength after use of LF-EMS was expected. The current trial however, showed no significant change in muscle strength. Muscle wasting, prevalent in many advanced heart failure patients,[34] could explain this observation. The chronic impairment of muscle tissue caused by heart failure affects the muscle and skin nerve receptors and hence contractility of the weakened muscle.[35] Participants with more functional leg muscles therefore, may have received greater stimulus to muscle tissue that others did for the same level of current intensity. This suggests that LF-EMS may not be effective for all advanced CHF patients.

Limitations

The current amplitude (mA) stimulus intensity that participants chose to use was a limitation to the study design. Participants were instructed to adhere to the 'maximum tolerable intensity' during LF-EMS sessions. Due to considerable individual differences in the subjective perception of discomfort associated with EMS, It is therefore likely that there was variability in the intensity that individuals received

Conclusion

As the majority of the predetermined feasibility criteria were met in this trial, a larger study into the effects of LF-EMS on advanced heart failure patients could be undertaken. This traditionally 'difficult to engage with' patient group could be recruited and adhere to LF-EMS therapy in sufficient numbers to

For peer review only - http://bmjopen.bmj.com/site/about/guidelines.xhtml

provide definitive data on its efficacy. The improvements seen in this study in 6MWD, and quality of life measures, showed a benefit with LF-EMS, although these were not statistically significant. Leg strength and physical activity levels showed no significant change. A longer intervention period than 8 weeks could be considered, to give participants more time to adjust to the intervention. More investigation is required to determine which CHF patients are unresponsive to LF-EMS due to severe muscle dysfunction.

A larger trial can be completed with this difficult population: whether the non-significant improvement in exercise capacity and quality of life found in this pilot study warrants further investigation in a larger trial is questionable.

Acknowledgements

We would like to thank Matthew Annals for his recruitment expertise and for administering the intervention to participants. We would also like to thank Josie Goodby for her assistance and patience during all assessments.

Author affiliations

1(Department of Cardiac Rehabilitation) University Hospitals Coventry & Warwickshire NHS Trust, UK
2University of Warwick (Clinical Trials Unit), UK
3 Liverpool John Moores University, UK
4 Cardiff Metropolitan University, UK
5 University Hospitals of Leicester NHS trust, UK
6 University of Coventry, UK

Author contributions

SE, GM and PB contributed to the conception of the work. SE, GM, PB, SS, HJ, RS, and TH contributed to the design of the work. SE and GM contributed to the acquisition, of the work. SE, GM, PB, SS, HJ, RS, and TH contributed to the, analysis, or interpretation of data for the work. SE and GM drafted the manuscript. PB, SS, HJ, RS, and TH critically revised the manuscript. All gave final approval and agree to be accountable for all aspects of work ensuring integrity and accuracy

Funding

This study was funded by a National Institute for Health Research (NIHR) Research for Patient Benefit (RfPB) award.

Declaration of Conflicting Interests

The Author(s) declare(s) that there is no conflict of interest

For peer review only

Ethics approval NRES Committee West Midlands - Coventry & Warwickshire13/WM/0240

Disclaimer

This article/paper/report presents independent research funded by the National Institute for Health Research (NIHR). The views expressed are those of the author(s) and not necessarily those of the NHS, the NIHR or the Department of Health.

Data sharing statement

All available data can be obtained by contacting the corresponding author:

stuart.ennis@uhcw.nhs.uk

References

1. Ambrosy, AP, Fonarow, GC, Butler J et al. The global health and economic burden of hospitilizations for heart failure: lessons learned from HHF registries. *J Am Coll Cardiol.* 2014; 63:1123-33

2. NICE 2010 guideline:CG108 Chronic heart failure (2010). available at: http://guidance.nice.org.uk/CG108NICEGuidance/pdf/English (accessed January 2016)

3. Kop WJ, Synowski SJ, Gottlieb SS. Depression in heart failure: biobehavioral mechanisms. *Heart Fail Clin*. 2011;7(1): 23-38.

4. Taylor RS, Sagar VA, Davies EJ et al. Exercise-based rehabilitation for heart failure. *Cochrane Database Syst Rev*. 2014;(4):CD003331. doi: 10.1002/14651858.CD003331.pub4

5. Piepoli M, Conraads V, Corrà U et al. Exercise training in heart failure: from theory to practice. A consensus document of the Heart Failure Association and the European Association for Cardiovascular Prevention and Rehabilitation. *Eur J Heart Fail*. 2011;13:347–357

For peer review only - http://bmjopen.bmj.com/site/about/guidelines.xhtml

6. Höllriegel R, Winzer EB, Linke A et al. Long-Term Exercise Training in Patients With Advanced Chronic Heart Failure: SUSTAINED BENEFITS ON LEFT VENTRICULAR PERFORMANCE AND EXERCISE CAPACITY *J Cardiopulm Rehabil Prev.* 2016;36(2):117-24

7. Piepoli MF, Davos C et al. ExTraMATCH Collaborative Exercise training meta-analysis of trials in patients with chronic heart failure (ExTraMATCH). *BMJ.* 2004;1(24): 328(7433):189

8. Sullach JA, Goldstein S. Use of beta-blockers in chronic heart failure. *Ann Med*. 2003; 35: 259-66.

9. Flather MD, Yusuf S, Køber L,et al. Long term ACE inhibitor therapy in patients with heart failure or left ventricular dysfunction: a systemic overview of the data from individual patients. ACE-Inhibitor Myocardial Infarction Collaborative Group *Lancet.* 2000;355:1578-81.

10. Smart NA, Dieberg G and Giallauria F. Functional electrical stimulation for chronic heart failure: a meta-analysis. *Int J Cardiol.* 2013;167(1): 80-86.

11. Nuhr, MJ, Pette D, Berger R et al. Beneficial effects of chronic low-frequency stimulation of thigh muscles in patients with advanced chronic heart failure. *Eur Heart J.* 2004;25(2): 136-143.

12. Dobsak P, Novakova M, Siegelova J et al. Low-frequency electrical stimulation increases muscle strength and improves blood supply in patients with chronic heart failure. *Circ J.* 2006;70(1): 75-82.

13. Crognale D, Vito GD, Grosset JF et al. (2013). Neuromuscular electrical stimulation can elicit aerobic exercise response without undue discomfort in healthy physically active adults. *J Strength Cond Res.* 2013; 27(1): 208-215.

14. Minogue CM, Caulfield BM, Lowery MM. Whole body oxygen uptake and evoked torque during sub-tetanic isometric electrical stimulation of the

For peer review only - http://bmjopen.bmj.com/site/about/guidelines.xhtml

quadriceps muscles in a single 30-minute session. *Arch Phys Med Rehabil*. 2014; 95(9):1750-8.

15. Banerjee P, Clark A, Witte K et al. Electrical stimulation of unloaded muscles causes cardiovascular exercise by increasing oxygen demand. *Eur J Cardiovasc Prev Rehabil.* 2005;12(5): 503-508.

16. Banerjee P, Caulfield B, Crowe L et al. Prolonged electrical muscle stimulation exercise improves strength, peak VO_2, and exercise capacity in patients with stable chronic heart failure. *J Card Fail.* 2009;15(4): 319-326.

17. Nieminen MS, Dickstein K, Fonseca C et al. The patient perspective: Quality of life in advanced heart failure with frequent hospitalisations. *Int J Cardiol.* 2015; 191:256-64.

18. Lancaster GA, Dodd S, Williamson PR. Design and analysis of pilot studies: recommendations for good practice. *J Eval Clin Pract.* 2004;10(2):307-12.

19. Thabane L, Ma J, Chu R, et al. A tutorial on pilot studies: The what, why and How. *BMC Medical Research Methodology.* 2010; (2)10:1.

20. Holland AE, Spruit MA, Troosters T et al. An official European Respiratory Society/American Thoracic Society technical standard: field walking tests in chronic respiratory disease. *Eur Respir J.* 2014;44(6): 1428-46.

21. Schaubert KL and Bohannon RW. Reliability and validity of three strength measures obtained from community-dwelling elderly persons *J Strength Cond Res.* 2005;19(3): 717-720

22. Rector TS and Cohn JN. Assessment of patient outcome with the Minnesota Living with Heart Failure questionnaire: reliability and validity during a randomized, double-blind, placebo-controlled trial of pimobendan. Pimobendan Multicenter Research Group. *Am Heart J.* 1992;124(4): 1017-1025.

For peer review only - http://bmjopen.bmj.com/site/about/guidelines.xhtml

23. Krueger, C. and Tian L. A comparison of the general linear mixed model and repeated measures ANOVA using a dataset with multiple missing data points. *Biol Res Nurs.* 2004;6(2): 151-157.

24. Alshurafa M, Briel M, Akl EA et al. Inconsistent definitions for intention-to-treat in relation to missing outcome data: systematic review of the methods literature. *PLoS One.* 2012; 7(11): e49163.

25. Borg G. Borg's Perceived exertion and pain scales. Champaign, IL, Human Kinetics.1998

26. The National Audit of Cardiac Rehabilitation—Annual Statistical Report 2014-2015. Available from http://www.cardiacrehabilitation.org.uk/docs/2007.pdf,

27. O'Connor CM, Whellan DJ, Lee KL et al. Efficacy and safety of exercise training in patients with chronic heart failure: HF-ACTION randomized controlled trial. *JAMA.* 2009; 301(14): 1439-1450.

28. Vivodtzev I, Rivard B, Gagnon P et al. Tolerance and physiological correlates of neuromuscular electrical stimulation in COPD: a pilot study. *PLoS One.* 2014; 9;9(5):e94850. doi: 10.1371/journal.pone.0094850

29. Jeon YH, Kraus SG, et al. The experience of living with chronic heart failure: a narrative review of qualitative studies. *BMC Health Serv Res*. 2010 24;10:77

30. Zugck C, Krüger C, Dürr S et al. Is the 6-minute walk test a reliable substitute for peak oxygen uptake in patients with dilated cardiomyopathy? *Eur Heart J*. 2000; 21(7):540-9

31. Täger T, Hanholz W, Cebola R et al. Minimal important difference for 6-minute walk test distances among patients with chronic heart failure. *Int J Cardiol*. 2014;176(1):94-8

For peer review only

32. Quittan M, Wiesinger GF, Sturm B et al. Improvement of thigh muscles by neuromuscular electrical stimulation in patients with refractory heart failure: a single-blind, randomized, controlled trial *Am J Phys Med Rehabil.* 2001;80(3): 206-214

33. Harris S, LeMaitre JP, Mackenzie G et al. A randomised study of home-based electrical stimulation of the legs and conventional bicycle exercise training for patients with chronic heart failure. *Eur Heart J.* 2003;24(9): 871-878.

34. Fülster S, Tacke M, Sandek A et al. Muscle wasting in patients with chronic heart failure: results from the studies investigating co-morbidities aggravating heart failure (SICA-HF). *Eur Heart J.* 2013;34(7): 512-9.

35. Rullman E, Andersson DC, Melin M et al. Modifications of skeletal muscle ryanodine receptor type 1 and exercise intolerance in heart failure. *J Heart Lung Transplant*. 2013;32(9):925-9. doi: 10.1016/j.healun.2013.06.026.

36. Dantas L, Vieira A, Siqueira A et al. Comparison between the effects of 4 different electrical stimulation current waveforms on isometric knee extension torque and perceived discomfort in healthy women. *Muscle Nerve.* 2015;51(1):76-82.

37. Medeiros F, Vieira A, Carregaro R et al. Skinfold thickness affects the isometric knee extension torque evoked by Neuromuscular Electrical Stimulation. *Braz J Phys Ther.* 2015;19(6): 466-72

For peer review only

For peer review only - http://bmjopen.bmj.com/site/about/guidelines.xhtml

CONSORT 2010 checklist of information to include when reporting a randomised trial*

Section/Topic	Item No	Checklist item	Reported on page No
**Title and abstract**			
	1a	Identification as a randomised trial in the title	1
	1b	Structured summary of trial design, methods, results, and conclusions (for specific guidance see CONSORT for abstracts)	2
**Introduction**			
Background and objectives	2a	Scientific background and explanation of rationale	3
	2b	Specific objectives or hypotheses	3
**Methods**			
Trial design	3a	Description of trial design (such as parallel, factorial) including allocation ratio	3
	3b	Important changes to methods after trial commencement (such as eligibility criteria), with reasons	n/a
Participants	4a	Eligibility criteria for participants	4
	4b	Settings and locations where the data were collected	4
Interventions	5	The interventions for each group with sufficient details to allow replication, including how and when they were actually administered	4,5
Outcomes	6a	Completely defined pre-specified primary and secondary outcome measures, including how and when they were assessed	5,6
	6b	Any changes to trial outcomes after the trial commenced, with reasons	n/a
Sample size	7a	How sample size was determined	
	7b	When applicable, explanation of any interim analyses and stopping guidelines	n/a
Randomisation:			
Sequence generation	8a	Method used to generate the random allocation sequence	4
	8b	Type of randomisation; details of any restriction (such as blocking and block size)	4
Allocation concealment mechanism	9	Mechanism used to implement the random allocation sequence (such as sequentially numbered containers), describing any steps taken to conceal the sequence until interventions were assigned	4
Implementation	10	Who generated the random allocation sequence, who enrolled participants, and who assigned participants to interventions	4
Blinding	11a	If done, who was blinded after assignment to interventions (for example, participants, care providers, those	4

For peer review only - http://bmjopen.bmj.com/site/about/guidelines.xhtml

		assessing outcomes) and how	
	11b	If relevant, description of the similarity of interventions	4
Statistical methods	12a	Statistical methods used to compare groups for primary and secondary outcomes	6
	12b	Methods for additional analyses, such as subgroup analyses and adjusted analyses	n/a
**Results**			
Participant flow (a diagram is strongly recommended)	13a	For each group, the numbers of participants who were randomly assigned, received intended treatment, and were analysed for the primary outcome	
	13b	For each group, losses and exclusions after randomisation, together with reasons	
Recruitment	14a	Dates defining the periods of recruitment and follow-up	7
	14b	Why the trial ended or was stopped	n/a
Baseline data	15	A table showing baseline demographic and clinical characteristics for each group	12
Numbers analysed	16	For each group, number of participants (denominator) included in each analysis and whether the analysis was by original assigned groups	10
Outcomes and estimation	17a	For each primary and secondary outcome, results for each group, and the estimated effect size and its precision (such as 95% confidence interval)	9,10,11,12
	17b	For binary outcomes, presentation of both absolute and relative effect sizes is recommended	n/a
Ancillary analyses	18	Results of any other analyses performed, including subgroup analyses and adjusted analyses, distinguishing pre-specified from exploratory	n/a
Harms	19	All important harms or unintended effects in each group (for specific guidance see CONSORT for harms)	11
**Discussion**			
Limitations	20	Trial limitations, addressing sources of potential bias, imprecision, and, if relevant, multiplicity of analyses	15
Generalisability	21	Generalisability (external validity, applicability) of the trial findings	14
Interpretation	22	Interpretation consistent with results, balancing benefits and harms, and considering other relevant evidence	14,15
**Other information**			
Registration	23	Registration number and name of trial registry	
Protocol	24	Where the full trial protocol can be accessed, if available	n/a
Funding	25	Sources of funding and other support (such as supply of drugs), role of funders	16

*We strongly recommend reading this statement in conjunction with the CONSORT 2010 Explanation and Elaboration for important clarifications on all the items. If relevant, we also recommend reading CONSORT extensions for cluster randomised trials, non-inferiority and equivalence trials, non-pharmacological treatments, herbal interventions, and pragmatic trials. Additional extensions are forthcoming: for those and for up to date references relevant to this checklist, see www.consort-statement.org.

For peer review only - http://bmjopen.bmj.com/site/about/guidelines.xhtml

BMJ Open

Randomised feasibility trial into the effects of low frequency electrical muscle stimulation in advanced heart failure patients

Journal:	*BMJ Open*
Manuscript ID	bmjopen-2017-016148.R1
Article Type:	Research
Date Submitted by the Author:	11-May-2017
Complete List of Authors:	Ennis, Stuart; University Hospitals Coventry and Warwickshire NHS Trust, Cardiac Rehabilitation; Cardiff Metropolitan University, Cardiff Physiology and Health<br>McGregor, Gordon; University Hospitals Coventry and Warwickshire NHS Trust, Cardiac Rehabilitation; Coventry University Faculty of Health and Life Sciences<br>Hamborg, Thomas; University of Warwick Warwick Medical School,<br>Jones, Helen; Liverpool John Moores University, sport and exercise sciences<br>Shave, Rob; Cardiff Metropolitan University, Physiology and Health<br>Singh, Sally; University Hospitals of Leicester NHS Trust, Cardiac/Pulmonary Rehabilitation<br>Banerjee, Prithwish; University Hospitals Coventry and Warwickshire NHS Trust, Department of Cardiology; Coventry University
**Primary Subject Heading**:	Sports and exercise medicine
Secondary Subject Heading:	Cardiovascular medicine, Rehabilitation medicine
Keywords:	electrical muscle stimulation, neuromuscular stimulation, cardiac rehabilitation, Heart failure < CARDIOLOGY

Note: The following files were submitted by the author for peer review, but cannot be converted to PDF. You must view these files (e.g. movies) online.

MAGIC PANTS Protocol V3 30 JUN 2014BMJ open.docm

SCHOLARONE™ Manuscripts

Randomised feasibility trial into the effects of low frequency electrical muscle stimulation in advanced heart failure patients

Stuart Ennis[1,4], Gordon McGregor[1,6], Thomas Hamborg [2], Helen Jones[3], Rob Shave[4], Sally J Singh[5,6] and Prithwish Banerjee[1,6].

For peer review only

Abstract .

Objective: Low Frequency Electrical Muscle Stimulation (LF-EMS) may have the potential to reduce breathlessness and increase exercise capacity in the chronic heart failure population who struggle to adhere to conventional exercise. The study's aim was to establish if a randomised controlled trial of LF-EMS was feasible.

Design and setting: Double blind (participants, outcome assessors), randomised study in a secondary care outpatient cardiac rehabilitation program.

Participants: Severe heart failure patients (New York Heart Association class III-IV) with left ventricular ejection fraction <40% documented by echocardiography were eligible.

Interventions: Participants were randomised (remotely by computer) to 8 weeks (5 x 60 mins per week) of either LF-EMS intervention (4Hz, continuous, n=30) or SHAM placebo (skin level stimulation only, n=30) of the quadriceps and hamstrings muscles. Participants used the LF-EMS straps at home and were supervised weekly

Outcome measures: Recruitment, adherence and tolerability to the intervention were measured during the trial as well as physiological outcomes (primary outcome: 6 minute walk, secondary outcomes: quadriceps strength, quality of life and physical activity).

Results: Sixty of 171 eligible participants (35.08%) were recruited to the trial. 12 (20%) of the 60 patients (4 LF-EMS, 8 SHAM) withdrew. Forty one patients (68.3 %) adhered to the protocol for at least 70% of the sessions**.** The physiological measures indicated no significant differences between groups in 6 minute walk distance, (P=0.13) and quality of life, (P=0.55) although both outcomes improved more with LF-EMS.

Conclusion: Severe heart failure patients can be recruited to and adhere to LF-EMS studies and find it tolerable. A larger Randomised Controlled Trial (RCT) in the advanced heart failure population is therefore feasible. However, the preliminary improvements in exercise capacity and quality of life were minimal and this should be considered when planning a larger trial.

Trial registration number: ISRCTN16749049

Strengths and Limitations

1. To our knowledge, this was the first study to evaluate the design of a study into LF-EMS in advanced (NYHA III-IV) heart failure patients
2. Analysis of recruitment, retention and adherence in this hard to reach group contributes useful knowledge to the heart failure literature on how practical exercise interventions could be implemented.
3. Participants were instructed to adhere to the 'maximum tolerable intensity' during LF-EMS sessions. Due to considerable individual differences in the subjective perception of discomfort associated with EMS, It is therefore likely that there was variability in the intensity that individuals received
4. This study had a small sample size, and was not powered or designed to assess the effects of LF-EMS in advanced heart failure. The findings should therefore be considered preliminary.

For peer review only - http://bmjopen.bmj.com/site/about/guidelines.xhtml

Introduction

Chronic Heart Failure (CHF) affects approximately 26 million people worldwide, [1] and is associated with a poor prognosis; 30- 40% of patients diagnosed with heart failure die within a year. [2] Patients in New York Heart Association (NYHA) class III/IV are unable to perform the simplest daily activities, become depressed and have a poor quality of life.[3]

Regular aerobic exercise reduces breathlessness and muscle dysfunction for individuals with CHF whilst improving exercise capacity.[4,5,6] According to the ExTraMATCH meta-analysis,[7] exercise training leads to a 35% relative reduction in mortality, similar to the effects of beta-blockers[8] and ACE inhibitors.[9] However, those with advanced CHF are often so limited that they are unable to gain the holistic benefits of exercise.[4,7]

Electrical Muscle Stimulation (EMS) may provide an alternative rehabilitative therapy for this group. In patients with mild to moderate CHF, EMS can improve muscle strength of the legs, exercise capacity and quality of life. [10,11,12] Low frequency (4-5Hz) electrical muscle stimulation (LF-EMS) produces shivering-like sub-tetanic muscle contractions that can stimulate an aerobic response equivalent to 51% of maximal oxygen uptake.[13] Therapeutic levels of aerobic exercise can thus be achieved passively by LF-EMS,[14] and it has been shown to be comfortable and well tolerated in healthy individuals and those with mild to moderate CHF. [15,16] However, the impact of LF-EMS in advanced heart failure (NYHA class III/IV) patients is currently unknown. As advanced heart failure patients have shown poor uptake and adherence to intervention studies,[17] a preliminary study was needed to determine the feasibility of LF-EMS in this patient cohort prior to the development of a large-scale definitive trial.

Based upon recommendations for good practice in the design of pilot and feasibility studies [18] this study was undertaken with the following aims: To (a) test the robustness of the study protocol for a potential future trial, (b) estimate rates of recruitment, consent and retention, (c) determine the tolerability of the LF-EMS intervention and the effectiveness of the sham placebo in the NYHA III/IV CHF population, and (d) gain initial estimates of the efficacy of LF-EMS for all potential primary outcomes. This can be used for sample size calculations in future substantive trials.

For peer review only

Methods

Experimental Design

This feasibility study used a double blind parallel group randomised control design. Participants were randomised to either LF-EMS or 'sham' placebo for a period of eight weeks and blinded to group allocation. Outcomes were assessed at baseline (pre randomisation), eight weeks and 20 weeks follow-up.

Recruitment and screening

Between October 2013 and March 2015, University Hospital Coventry and Warwickshire, (UHCW) Hospital NHS Trust heart failure clinics lists were screened for patients fulfilling the eligibility criteria for the study. Sixty eligible participants were recruited. The study conformed to the Declaration of Helsinki and was approved by the local NHS Ethics Committee. All participants provided written informed consent.

Randomisation

The trial statistician, in conjunction with Warwick Clinical Trials Unit generated the randomisation sequence remotely (by computer) using permuted block randomisation. Group allocation was concealed from outcomes assessors and participants.

Participants

Male and female adults, >18 years old, with stable CHF, documented by echocardiography of left ventricular systolic dysfunction (ejection fraction < 40%) were eligible for the study. All participants had New York Heart Association (NYHA) functional class III-IV symptoms as judged by an experienced heart failure cardiologist. Participants were required to be medically stable, defined as the absence of hospital admission or alterations in medical therapy within the preceding two weeks. Exclusion criteria for safety and practical reasons were: (1) presence of implantable cardiac devices, (2) serious cardiac arrhythmias,(3) neurological disorders or previous stroke significant enough to limit exercise, (4) orthopaedic problems that prevented walking, (5) neuromuscular disease, (6) dementia or (7) a mid-thigh circumference of more than 50cm (due to the size of the LF-EMS straps).

LF-EMS Stimulation

The LF-EMS equipment (Biomedical Research Limited, Galway, Ireland) consisted of a pair of neoprene straps containing built-in adhesive gel electrodes. The equipment is CE marked under the European Medical Device Directive. The stimulator current waveform was designed to produce rhythmical contractions in the leg muscle groups occurring at a pulse frequency of 4-5Hz (pulse width: 620μs). The maximum peak output pulse current used was 140mA.

LF-EMS intervention

Participants used the LF-EMS or sham placebo for one hour, five times a week, for eight consecutive weeks. Of the five hourly sessions per week, four were

For peer review only - http://bmjopen.bmj.com/site/about/guidelines.xhtml

completed unsupervised in the participant's own home. The remaining session was conducted in a cardiac rehabilitation outpatient setting under the supervision of an exercise physiologist. The LF-EMS technology was retrospectively interrogated (i.e. at the weekly supervised sessions) to report date, frequency, duration and stimulation intensity.

'Sham' Placebo intervention

In the sham arm of the study, participants were provided with identical straps and electrodes. In contrast to the LF-EMS group the controller was programmed to deliver a very low level of stimulation (Frequency: 99Hz, pulse width: 150μs, maximum current amplitude: 7.3mA). This provided sensory input to the skin surface but little or no muscle activation. Participants in the sham group had the same induction, supervision and follow-up as the intervention arm.

Feasibility criteria

In relation to the design of pilot and feasibility studies, Thabane et al,[19] recommends stipulating criteria for success '*a priori*'. The feasibility criteria were:

1. *Recruitment rate* – At least 40% of eligible participants recruited to the trial

2. *Retention* – no more than 33% of participants drop out during the intervention period.

3. *Adherence* – 66% of participants tolerate the intervention and adhere to the protocol for ≥70% of the intervention period.

4. *Placebo efficacy*- Participants would be able to guess their group allocation no more often than would be expected by chance.

Primary outcome

Six Minute Walk Test (6MWT).

The 6MWT was conducted in accordance with the American Thoracic Society (ATS) guidelines.[20] Participants were instructed to walk as far as possible in six minutes along a 30m, flat, obstacle free corridor, turning 180 degrees at the end of every 30m. Standardised instructions and verbal encouragement were given.

Secondary outcomes

Isometric muscle strength

A hand held dynamometer (MicroFET2 Torque/Force indicator, Hoggan Health Industries, Utah, US) validated for assessing functional leg strength in elderly populations was used.[21] Participants sat in an elevated chair and were instructed to maximally extend the knee while the assessor provided an equal and opposite resistive force, against the lower shin. Mean force generated was measured in Newtons .

For peer review only - http://bmjopen.bmj.com/site/about/guidelines.xhtml

Quality of Life: Minnesota Living with Heart Failure Questionnaire (MLHFQ)

The MLWHF questionnaire is a disease validated questionnaire,[22] that has been extensively used in heart failure studies. Questionnaire scores range from 0 to 105, with higher scores reflecting lower Quality of life. Participants were asked to answer each question based on their perception of health in the week previous to testing.

Physical Activity levels

Physical activity levels were measured by the Bodymedia© SenseWear Pro3 Armband. The multi-plane accelerometer was worn continuously for the seven days prior to testing to determine Total Energy Expenditure (TEE) per 24hr period was used as the main indicator of physical activity.

Safety: Blood test

Venous blood samples were taken at baseline, four weeks and eight weeks to assess creatine kinase (CK), urea, and electrolytes. Participants would discontinue the trial if levels exceeded the upper limit of normal reference ranges

Data analysis

Data analyses for the feasibility objectives of this study were descriptive, based on the pre-determined levels specified above. Confidence intervals (set at 95%) were calculated for all secondary outcome measures in both groups and paired two-sample t-test conducted for between group comparisons. Intent-to-treat (ITT) analysis was employed in this study as is recommended for clinical trials.[24]

For peer review only

For peer review only - http://bmjopen.bmj.com/site/about/guidelines.xhtml

Results

Feasibility criteria outcomes

Recruitment

There were 171 eligible participants identified in the Coventry and Warwickshire area from November 2013 - April 2015. Sixty of 171 eligible participants (35.08%) were recruited to the trial. Participants were randomised and started on the trial during this period and were followed up until data collection finished in August 2015. Participant characteristics are presented in Table 1.

For peer review only

Table 1. Baseline demographic and clinical characteristics of the LF-EMS and sham placebo groups. Data presented as mean ± SD or absolute number and percent.

**Demographics**	**LF-EMS (n-30)**	**Sham (n=30)**
n Male	20 (66%)	22 (73%)
Age (yrs)	66.5 ± 7.8	66.8 ± 13.5
Body Mass Index (kg/m^2)	30.1 ± 4.9	27.8 ± 4.8
**Comorbidities**		
Prev MI/PCI/CABG	17 (56%)	11 (36%)
Diabetes	12 (40%)	10 (33%)
COPD	9 (30%)	8 (26%)
AF	20 (66%)	16 (53%)
Hypertension	13 (43%)	10 (33%
CKD	5 (16%)	13 (43%)
**Clinical**		
NT-pro-BNP (pg/mL)	3086 ± 3746	2046 ± 2545
Creatinine (μmol/L)	108 ± 49	113 ± 39
LVEF %	39 ± 11*	22 ± 12**
BP_{sys} (mmHg)	118 ± 16	126 ± 17
BP_{dia} (mmHg)	69 ± 9	74 ± 14
NYHA III	24 (80%)	22 (73%)
NYHA IV	6 (20%)	8 (26%)

NT-pro-BNP (pg/mL),N-terminal pro B-type natriuretic peptide; LVEF; left ventricular ejection fraction; BP_{sys} (mmHg), systolic blood pressure; BP_{dia} (mmHg), diastolic blood pressure; NYHA, New York Heart association; MI, myocardial infarction; PCI, percutaneous coronary intervention; CABG, coronary artery bypass graft surgery; COPD, chronic obstructive pulmonary disease; AF, atrial fibrillation; CKD, chronic kidney disease;

*n=10.Ejection fraction could not be accurately assessed in all patients due to poor body habitus/atrial fibrillation. An experienced cardiac sonographer made an 'eyeball' assessment of poor left ventricular function for all other participants

**n=5. See previous comments.

For peer review only - http://bmjopen.bmj.com/site/about/guidelines.xhtml

Retention

Twelve of the 60 participants (4 LF-EMS, 8 sham) (20%) withdrew and did not finish the intervention period (See Fig 1). Of these, only three found the intervention intolerable (1 LF-EMS, 2 sham). Other reasons for dropout were: deterioration in health (n= 6) family problems (n=2) and implantation of a cardioverter defibrillator (ICD) (n=1). Only 22 (45%) of those completing the intervention period returned for follow-up testing at 20 weeks. Reasons for non-follow-up were: deterioration in health (n=9), excluded due to implantation of cardiac resynchronisation therapy device (n=2), declined to take part without further explanation (n=13), and could not be contacted after repeated attempts (n= 3).

For peer review only

For peer review only - http://bmjopen.bmj.com/site/about/guidelines.xhtml

For peer review only

Figure 1. Flow diagram of a single centre blinded parallel group randomised feasibility trial of electrical muscle stimulation versus sham placebo in severe heart failure patients.

Adherence

Forty one (85.4 %) of the 48 participants (22-LF-EMS, 19-SHAM) who completed the intervention period (68.3% of the total sample) adhered to the strict protocol for the majority (>70%) of the eight weeks. Interrogation of the LF-EMS controllers revealed that participants in the LF-EMS group became more tolerant to the intervention; mean stimulation intensity increased from 57.79mA (95%CI: 51.16 to 64.42) during week 1 of the study to 84.86mA (95%CI: 75.44 to 94.28) by week 8, an improvement of 46.5%.

'Sham' Placebo

The sham placebo for the study appeared to be convincing as only 61% of participants guessed their treatment group correctly. The 95% confidence interval for the proportion of participants guessing correctly was (46% to 74%) and thus not significantly different from 50% which would be expected by chance. Furthermore, participants demonstrated an inclination to guess that they were randomised to LF-EMS regardless of group allocation.

Safety

No abnormalities were detected in CK, urea or electrolytes taken before, during or after the study. Likewise, no adverse events due to the intervention were recorded in either group.

Primary outcome- 6-minute walk test

Non-significant improvements after LF-EMS (8 week time point) and sham groups were observed in 6 MWD with a mean increase from baseline of 24m in the LF-EMS group and 9m in the sham group respectively (Table 2.)

Secondary Outcomes

Table 2 shows the mean values of outcome measures at each time point. There were no significant differences between groups in the change from baseline for any of the secondary outcome variables (Table 3). There was a non-significant improvement in quality of life in both groups.

For peer review only

Table 2: Outcome measurements – Time point averages and 95% confidence intervals (CI)

Outcome	Time point	LF-EMS	Sham
Mean 6 MWD (metres) [95% CI]	Baseline	283 [237 – 328]	290 [243 – 337]
	(n)	29	29
	8 weeks	312 [262 – 362]	318 [270 – 365]
	(n)	26	22
	20 weeks	257 [173 – 342]	226 [126 – 325]
	(n)	12	10
(Mean leg strength (newtons) [95% CI]	Baseline	234.3 [196.5 – 272.]	297.5 [253 – 342]
	(n)	29	29
	8 weeks	224.9 [187.5 – 262.3]	321 [267.8 – 374.3]
	(n)	25	22
	20 weeks	181.6 [131.7 – 231.5]	207.1 [148.6 – 265.7]
	(n)	11	10
Mean QoL (score) [95% CI]	Baseline	53.1 [42.7 – 63.5]	50 [40 – 60.1]
	(n)	28	29
	8 weeks	43.9 [34.2 – 53.5]	43.1 [30.9 – 55.3]
	(n)	25	22
	20 weeks	51.7 [31.6 – 71.8]	37.0 [16.9 – 57]
	(n)	12	10
Mean TEE (joules) *[95% CI]*	Baseline	63,438 [56,170 – 70,705]	65,371 [59675 – 71,067]
	(n)	25	27
	8 weeks	59,783 [51,094 – 68,471]	59,687 [50,630 – 68,745]
	(n)	19	17
	20 weeks	61,878 [53,345 – 70,410]	63,541 [55,795 – 71,287]
	(n)	7	6

6 MWD, 6 minute walk distance, QoL, quality of life; TEE, Total Energy Expenditure

For peer review only

For peer review only - http://bmjopen.bmj.com/site/about/guidelines.xhtml

For peer review only

For peer review only - http://bmjopen.bmj.com/site/about/guidelines.xhtml

Table 3: -Changes from baseline averages and 95% confidence intervals (CI)

Outcome	Time point	LF-EMS	Sham	p-value
Mean 6 MWD (metres) [95% CI]	Baseline to 8 weeks	24 [9 – 40]	9 [-4 – 22]	0.1366
	(n)	26	22	
	Baseline to 20 weeks	0 [-32 – 31]	-26.30 [-63 – 11]	0.2409
	(n)	12	10	
(Mean leg strength (newtons) [95% CI]	Baseline to 8 weeks	-9.2 [-28.9 – 10.5]	6.0 [-19.3 – 31.4]	0.3244
	(n)	25	22	
	Baseline to 20 weeks	-43.4 [-78.7 – -8.2]	-74.1 [-116.3 – -31.9]	0.2223
	(n)	11	10	
Mean QoL (score) [95% CI]	Baseline to 8 weeks	-7.6 [-15.5 – 0.3]	-4.7 [-10.5 – 1.0]	0.5505
	(n)	25	22	
	Baseline to 20 weeks	1.5 [-12.5 – 15.7]	-14.0 [-34 – 6]	0.1610
	(n)	12	10	
Mean TEE (joules) [95% CI]	Baseline to 8 weeks	-4635 [-3963 – 4692]	-8168 [-14,342 – -1995]	0.5108
	(n)	19	17	
	Baseline to 20 weeks	1686 [-6435 – 9809]	4177 [-7695 – 16,050]	0.6634
	(n)	7	6	

6 MWD, 6 minute walk distance; QoL, quality of life; TEE, Total Energy Expenditure

For peer review only

For peer review only - http://bmjopen.bmj.com/site/about/guidelines.xhtml

Sample size calculation

The point estimate from the study and the upper CI limit of this estimate were calculated. The upper CI limit was used for the sample size calculation. For detecting the observed difference of 13.4 metres in this study a sample size of 240 patients per group would be required. However, a recent study [25] suggested that the minimal clinically important difference for 6MWD is 36 metres in mild-moderate CHF patients. The clinical benefit of the effect size in this study should be considered before proceeding with a larger trial

For peer review only

Discussion

The predetermined criteria for proceeding to a larger trial were achieved for dropout (20%), adherence (68.3%) and sham placebo efficacy (61.53% participants guessed correctly). However, only 35.06% of eligible patients were recruited, below the target of 40%. Initial outcome measures revealed no significant difference between intervention and placebo groups, although there was a non-significant improvement in 6MWD and quality of life after LF-EMS.

Feasibility outcomes

Recruitment

Percentage uptake (35.06%) of eligible patients in the study was below the predetermined criteria of 40%. This is similar to the poor uptake of conventional cardiac rehabilitation (CR) nationally in the UK: less than 40% of eligible post myocardial infarction patients and those undergoing elective percutaneous coronary interventions (PCI) accessed CR in the most recent National Audit of Cardiac Rehabilitation.[26]

Retention/adherence/tolerance

One strength of this study is the good level of adherence (68.3%) and retention (80%) compared with other clinical studies; In the HF-ACTION trial,[27] only 40% of patients in the exercise group (n=1159) reported adherence to recommended training volumes after three months. This may have been because of the ease of independent use at home of LF-EMS, in combination with the weekly supervised sessions with an exercise physiologist The patients recruited in the present trial were more debilitated yet they engaged more with LF-EMS than those in the HF-ACTION trial, [27] suggesting that LF-EMS maybe more acceptable to this population than conventional exercise. The dropout at 3 months follow-up was lower than expected due to ill health, device implantation and apathy. Strategies to prevent dropout such as combining assessment with clinical patient appointments would need to be developed in a larger trial. Tolerance to the LF-EMS intervention improved during the study. Mean current intensity increased by 46% from week one to week eight. This tolerance effect is in keeping with an earlier study by Crognale, et al,[13] that showed a 20% increase in healthy active adults. The active adults tolerated higher absolute stimulation levels than in this study, both before and after habituation, suggesting that advanced CHF patients are subjectively less tolerant to LF-EMS than a healthy population. Vivodtzev and colleagues,[28] examined factors determining tolerance of EMS in pulmonary patients. The study reported that lower tolerance to EMS was associated with greater severity of condition, fat free mass and inflammatory response. It is possible that the same is true in the CHF population but more research is needed to confirm this.

Outcome measures

For peer review only - http://bmjopen.bmj.com/site/about/guidelines.xhtml

Baseline 6MWD was higher in our study sample than in other advanced heart failure studies.[29] This may have been due to high variability because of a few outliers in each group. This reflects the subjective nature of the NYHA classification system. However, signs and symptoms of advanced heart failure were primarily the eligibility criteria for this study and not 6MWD. In addition, the ≤300-m distance cutoff (below which our baseline mean falls) is often cited, as prognostically important and reflective of advanced disease in many investigations.[30,31,32] The non-significant improvements in exercise capacity as measured by 6 minute walk were smaller than those in a meta-analysis of EMS in heart failure patients by Smart, Dieberg and Gialluria.[10] These authors reported a combined improvement in 6MWD of 46.9m vs usual care or placebo, compared to the effect size of 13.2m in this study. However, patients in this study were more symptomatic than those included in the meta-analysis,[10] and thus had a lower baseline exercise capacity (286m vs 342m.) Nevertheless, the relative increase (5%) in walk distance of participants in the LF-EMS group is within the measurement error associated with this test,[30] and probably should not be considered clinically significant.[31] The extrapolation from these results that severe CHF patients are beyond help from EMS maybe premature; a longer training period maybe required to show meaningful changes in exercise capacity, particularly as some participants took longer to tolerate meaningful EMS intensities than others.

Quality of life (MLHFQ) improved in both groups after the intervention. This may, in part, relate to the psychosocial benefits of engaging with researchers regularly in the cardiac rehabilitation facility.[29] The placebo effect of both interventions and its influence on patients' perception of well-being should not be underestimated.

Based on previous research by Banerjee et al,[15,16] and numerous high frequency EMS studies,[32,33,12] improvement in leg strength after use of LF-EMS was expected. The current trial however, showed no significant change in muscle strength. Muscle wasting, prevalent in many advanced heart failure patients,[34] could explain this observation. The chronic impairment of muscle tissue caused by heart failure affects the muscle and skin nerve receptors and hence contractility of the weakened muscle.[35] Participants with more functional leg muscles therefore, may have received greater stimulus to muscle tissue that others did for the same level of current intensity. This suggests that LF-EMS may not be effective for all advanced CHF patients.

Limitations

The sample for this study was small as is recommended for feasability studies[19] and this limits the external validity of our findings. The current amplitude (mA) stimulus intensity that participants chose to use was a limitation to the study design. Participants were instructed to adhere to the 'maximum tolerable intensity' during LF-EMS sessions. Due to considerable individual differences in

For peer review only - http://bmjopen.bmj.com/site/about/guidelines.xhtml

the subjective perception of discomfort associated with EMS, It is therefore likely that there was variability in the intensity that individuals received

Conclusion

As the majority of the predetermined feasibility criteria were met in this trial, a larger study into the effects of LF-EMS on advanced heart failure patients could be undertaken. This traditionally 'difficult to engage with' patient group could be recruited and adhere to LF-EMS therapy in sufficient numbers to provide definitive data on its efficacy. The improvements seen in this study in 6MWD, and quality of life measures, showed a benefit with LF-EMS, although these were not statistically significant. Leg strength and physical activity levels showed no significant change. A longer intervention period than 8 weeks could be considered, to give participants more time to adjust to the intervention. More investigation is required to determine which CHF patients are unresponsive to LF-EMS due to severe muscle dysfunction.

A larger trial can be completed with this difficult population: whether the non-significant improvement in exercise capacity and quality of life found in this pilot study would be improved upon in a larger pragmatic trial should be considered.

Acknowledgements

We would like to thank Matthew Annals for his recruitment expertise and for administering the intervention to participants. We would also like to thank Josie Goodby for her assistance and patience during all assessments.

Author affiliations

1 (Department of Cardiac Rehabilitation) University Hospitals Coventry & Warwickshire NHS Trust, UK
2 University of Warwick (Clinical Trials Unit), UK
3 Liverpool John Moores University, UK
4 Cardiff Metropolitan University, UK
5 University Hospitals of Leicester NHS trust, UK
6 University of Coventry, UK

Author contributions

SE, GM and PB contributed to the conception of the work. SE, GM, PB, SS, HJ, RS, and TH contributed to the design of the work. SE and GM contributed to the acquisition, of the work. SE, GM, PB, SS, HJ, RS, and TH contributed to the, analysis, or interpretation of data for the work. SE and GM drafted the manuscript. PB, SS, HJ, RS, and TH critically revised the manuscript. All gave final approval and agree to be accountable for all aspects of work ensuring integrity and accuracy

For peer review only - http://bmjopen.bmj.com/site/about/guidelines.xhtml

For peer review only

Funding

This study was funded by a National Institute for Health Research (NIHR) Research for Patient Benefit (RfPB) award.

Declaration of Conflicting Interests

The Author(s) declare(s) that there is no conflict of interest

Ethics approval NRES Committee West Midlands - Coventry & Warwickshire13/WM/0240

Disclaimer

This article/paper/report presents independent research funded by the National Institute for Health Research (NIHR). The views expressed are those of the author(s) and not necessarily those of the NHS, the NIHR or the Department of Health.

Data sharing statement

All available data can be obtained by contacting the corresponding author:

stuart.ennis@uhcw.nhs.uk

References

1. Ambrosy, AP, Fonarow, GC, Butler J et al. The global health and economic burden of hospitilizations for heart failure: lessons learned from HHF registries. *J Am Coll Cardiol.* 2014; 63:1123-33

2. NICE 2010 guideline:CG108 Chronic heart failure (2010). available at: http://guidance.nice.org.uk/CG108NICEGuidance/pdf/English (accessed January 2016)

3. Kop WJ, Synowski SJ, Gottlieb SS. Depression in heart failure: biobehavioral mechanisms. *Heart Fail Clin*. 2011;7(1): 23-38.

4. Taylor RS, Sagar VA, Davies EJ et al. Exercise-based rehabilitation for heart failure. *Cochrane Database Syst Rev*. 2014;(4):CD003331. doi: 10.1002/14651858.CD003331.pub4

For peer review only - http://bmjopen.bmj.com/site/about/guidelines.xhtml

5. Piepoli M, Conraads V, Corrà U et al. Exercise training in heart failure: from theory to practice. A consensus document of the Heart Failure Association and the European Association for Cardiovascular Prevention and Rehabilitation. *Eur J Heart Fail*. 2011;13:347–357

6. Höllriegel R, Winzer EB, Linke A et al. Long-Term Exercise Training in Patients With Advanced Chronic Heart Failure: SUSTAINED BENEFITS ON LEFT VENTRICULAR PERFORMANCE AND EXERCISE CAPACITY *J Cardiopulm Rehabil Prev.* 2016;36(2):117-24

7. Piepoli MF, Davos C et al. ExTraMATCH Collaborative Exercise training meta-analysis of trials in patients with chronic heart failure (ExTraMATCH). *BMJ.* 2004;1(24): 328(7433):189

8. Sullach JA, Goldstein S. Use of beta-blockers in chronic heart failure. *Ann Med*. 2003; 35: 259-66.

9. Flather MD, Yusuf S, Køber L,et al. Long term ACE inhibitor therapy in patients with heart failure or left ventricular dysfunction: a systemic overview of the data from individual patients. ACE-Inhibitor Myocardial Infarction Collaborative Group *Lancet.* 2000;355:1578-81.

10. Smart NA, Dieberg G and Giallauria F. Functional electrical stimulation for chronic heart failure: a meta-analysis. *Int J Cardiol.* 2013;167(1): 80-86.

11. Nuhr, MJ, Pette D, Berger R et al. Beneficial effects of chronic low-frequency stimulation of thigh muscles in patients with advanced chronic heart failure. *Eur Heart J.* 2004;25(2): 136-143.

12. Dobsak P, Novakova M, Siegelova J et al. Low-frequency electrical stimulation increases muscle strength and improves blood supply in patients with chronic heart failure. *Circ J.* 2006;70(1): 75-82.

For peer review only - http://bmjopen.bmj.com/site/about/guidelines.xhtml

13. Crognale D, Vito GD, Grosset JF et al. (2013). Neuromuscular electrical stimulation can elicit aerobic exercise response without undue discomfort in healthy physically active adults. *J Strength Cond Res.* 2013; 27(1): 208-215.

14. Minogue CM, Caulfield BM, Lowery MM. Whole body oxygen uptake and evoked torque during sub-tetanic isometric electrical stimulation of the quadriceps muscles in a single 30-minute session. *Arch Phys Med Rehabil*. 2014; 95(9):1750-8.

15. Banerjee P, Clark A, Witte K et al. Electrical stimulation of unloaded muscles causes cardiovascular exercise by increasing oxygen demand. *Eur J Cardiovasc Prev Rehabil.* 2005;12(5): 503-508.

16. Banerjee P, Caulfield B, Crowe L et al. Prolonged electrical muscle stimulation exercise improves strength, peak VO_2, and exercise capacity in patients with stable chronic heart failure. *J Card Fail.* 2009;15(4): 319-326.

17. Nieminen MS, Dickstein K, Fonseca C et al. The patient perspective: Quality of life in advanced heart failure with frequent hospitalisations. *Int J Cardiol.* 2015; 191:256-64.

18. Lancaster GA, Dodd S, Williamson PR. Design and analysis of pilot studies: recommendations for good practice. *J Eval Clin Pract.* 2004;10(2):307-12.

19. Thabane L, Ma J, Chu R, et al. A tutorial on pilot studies: The what, why and How. *BMC Medical Research Methodology.* 2010; (2)10:1.

20. Holland AE, Spruit MA, Troosters T et al. An official European Respiratory Society/American Thoracic Society technical standard: field walking tests in chronic respiratory disease. *Eur Respir J.* 2014;44(6): 1428-46.

21. Schaubert KL and Bohannon RW. Reliability and validity of three strength measures obtained from community-dwelling elderly persons *J Strength Cond Res.* 2005;19(3): 717-720

For peer review only - http://bmjopen.bmj.com/site/about/guidelines.xhtml

22. Rector TS and Cohn JN. Assessment of patient outcome with the Minnesota Living with Heart Failure questionnaire: reliability and validity during a randomized, double-blind, placebo-controlled trial of pimobendan. Pimobendan Multicenter Research Group. *Am Heart J.* 1992;124(4): 1017-1025.

23. Krueger, C. and Tian L. A comparison of the general linear mixed model and repeated measures ANOVA using a dataset with multiple missing data points. *Biol Res Nurs.* 2004;6(2): 151-157.

24. Alshurafa M, Briel M, Akl EA et al. Inconsistent definitions for intention-to-treat in relation to missing outcome data: systematic review of the methods literature. *PLoS One.* 2012; 7(11): e49163.

25. Täger T, Hanholz W, Cebola R et al. Minimal important difference for 6-minute walk test distances among patients with chronic heart failure. *Int J Cardiol*. 2014;176(1):94-8

26. The National Audit of Cardiac Rehabilitation—Annual Statistical Report 2014-2015. Available from http://www.cardiacrehabilitation.org.uk/docs/2007.pdf,

27. O'Connor CM, Whellan DJ, Lee KL et al. Efficacy and safety of exercise training in patients with chronic heart failure: HF-ACTION randomized controlled trial. *JAMA.* 2009; 301(14): 1439-1450.

28. Vivodtzev I, Rivard B, Gagnon P et al. Tolerance and physiological correlates of neuromuscular electrical stimulation in COPD: a pilot study. *PLoS One.* 2014; 9;9(5):e94850. doi: 10.1371/journal.pone.0094850

29. Reeves GR, Whellan DJ, O'Connor CM et al. A Novel Rehabilitation Intervention for Older Patients With Acute Decompensated Heart Failure *JACC: Heart Failure* 2017, 613; DOI: 10.1016/j.jchf.2016.12.019

For peer review only - http://bmjopen.bmj.com/site/about/guidelines.xhtml

30. Guazzi M, Dickstein K, Vicenzi R and Arena R. Six-Minute Walk Test and Cardiopulmonary Exercise Testing in Patients With Chronic Heart Failure. *Circulation: Heart Failure*. 2009; 2:549-555.

31. Arslan S, Erol MK, Gundogdu F et al. Prognostic value of 6-minute walk test in stable outpatients with heart failure. *Tex Heart Inst J.* 2007; **34**: 166–169.

32. Rostagno C, Olivo G, Comeglio M, et al. Prognostic value of 6-minute walk corridor test in patients with mild to moderate heart failure: comparison with other methods of functional evaluation.2003 *Eur J Heart Fail.* **5**: 247–252.

33. Zugck C, Krüger C, Dürr S et al. Is the 6-minute walk test a reliable substitute for peak oxygen uptake in patients with dilated cardiomyopathy? *Eur Heart J*. 2000; 21(7):540-9

34. Jeon YH, Kraus SG, et al. The experience of living with chronic heart failure: a narrative review of qualitative studies. *BMC Health Serv Res*. 2010 24;10:77

35. Quittan M, Wiesinger GF, Sturm B et al. Improvement of thigh muscles by neuromuscular electrical stimulation in patients with refractory heart failure: a single-blind, randomized, controlled trial *Am J Phys Med Rehabil.* 2001;80(3): 206-214

36. Harris S, LeMaitre JP, Mackenzie G et al. A randomised study of home-based electrical stimulation of the legs and conventional bicycle exercise training for patients with chronic heart failure. *Eur Heart J.* 2003;24(9): 871-878.

37. Fülster S, Tacke M, Sandek A et al. Muscle wasting in patients with chronic heart failure: results from the studies investigating co-morbidities aggravating heart failure (SICA-HF). *Eur Heart J*. 2013;34(7): 512-9.

For peer review only

For peer review only - http://bmjopen.bmj.com/site/about/guidelines.xhtml

38. Rullman E, Andersson DC, Melin M et al. Modifications of skeletal muscle ryanodine receptor type 1 and exercise intolerance in heart failure. *J Heart Lung Transplant*. 2013;32(9):925-9. doi: 10.1016/j.healun.2013.06.026.

For peer review only

For peer review only - http://bmjopen.bmj.com/site/about/guidelines.xhtml

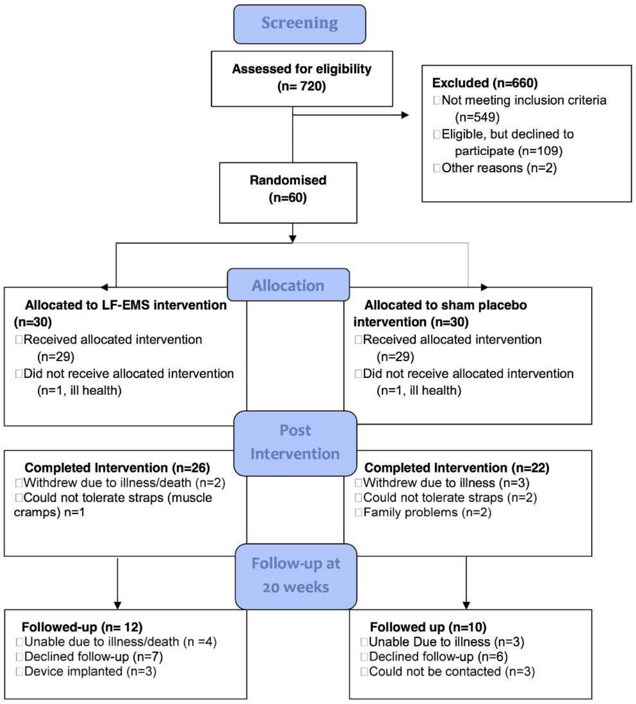

Figure 1. Flow diagram of a single centre blinded parallel group randomised feasibility trial of electrical muscle stimulation versus sham placebo in severe heart failure patients.

209x297mm (300 x 300 DPI)

CONSORT 2010 checklist of information to include when reporting a randomised trial*

Section/Topic	Item No	Checklist item	Reported on page No
**Title and abstract**			
	1a	Identification as a randomised trial in the title	1
	1b	Structured summary of trial design, methods, results, and conclusions (for specific guidance see CONSORT for abstracts)	2
**Introduction**			
Background and objectives	2a	Scientific background and explanation of rationale	3
	2b	Specific objectives or hypotheses	3
**Methods**			
Trial design	3a	Description of trial design (such as parallel, factorial) including allocation ratio	3
	3b	Important changes to methods after trial commencement (such as eligibility criteria), with reasons	n/a
Participants	4a	Eligibility criteria for participants	4
	4b	Settings and locations where the data were collected	4
Interventions	5	The interventions for each group with sufficient details to allow replication, including how and when they were actually administered	4,5
Outcomes	6a	Completely defined pre-specified primary and secondary outcome measures, including how and when they were assessed	5,6
	6b	Any changes to trial outcomes after the trial commenced, with reasons	n/a
Sample size	7a	How sample size was determined	
	7b	When applicable, explanation of any interim analyses and stopping guidelines	n/a
Randomisation:			
Sequence generation	8a	Method used to generate the random allocation sequence	4
	8b	Type of randomisation; details of any restriction (such as blocking and block size)	4
Allocation concealment mechanism	9	Mechanism used to implement the random allocation sequence (such as sequentially numbered containers), describing any steps taken to conceal the sequence until interventions were assigned	4
Implementation	10	Who generated the random allocation sequence, who enrolled participants, and who assigned participants to interventions	4
Blinding	11a	If done, who was blinded after assignment to interventions (for example, participants, care providers, those	4

For peer review only - http://bmjopen.bmj.com/site/about/guidelines.xhtml

		assessing outcomes) and how	
	11b	If relevant, description of the similarity of interventions	4
Statistical methods	12a	Statistical methods used to compare groups for primary and secondary outcomes	6
	12b	Methods for additional analyses, such as subgroup analyses and adjusted analyses	n/a
**Results**			
Participant flow (a diagram is strongly recommended)	13a	For each group, the numbers of participants who were randomly assigned, received intended treatment, and were analysed for the primary outcome	
	13b	For each group, losses and exclusions after randomisation, together with reasons	
Recruitment	14a	Dates defining the periods of recruitment and follow-up	7
	14b	Why the trial ended or was stopped	n/a
Baseline data	15	A table showing baseline demographic and clinical characteristics for each group	12
Numbers analysed	16	For each group, number of participants (denominator) included in each analysis and whether the analysis was by original assigned groups	10
Outcomes and estimation	17a	For each primary and secondary outcome, results for each group, and the estimated effect size and its precision (such as 95% confidence interval)	9,10,11,12
	17b	For binary outcomes, presentation of both absolute and relative effect sizes is recommended	n/a
Ancillary analyses	18	Results of any other analyses performed, including subgroup analyses and adjusted analyses, distinguishing pre-specified from exploratory	n/a
Harms	19	All important harms or unintended effects in each group (for specific guidance see CONSORT for harms)	11
**Discussion**			
Limitations	20	Trial limitations, addressing sources of potential bias, imprecision, and, if relevant, multiplicity of analyses	15
Generalisability	21	Generalisability (external validity, applicability) of the trial findings	14
Interpretation	22	Interpretation consistent with results, balancing benefits and harms, and considering other relevant evidence	14,15
**Other information**			
Registration	23	Registration number and name of trial registry	
Protocol	24	Where the full trial protocol can be accessed, if available	n/a
Funding	25	Sources of funding and other support (such as supply of drugs), role of funders	16

*We strongly recommend reading this statement in conjunction with the CONSORT 2010 Explanation and Elaboration for important clarifications on all the items. If relevant, we also recommend reading CONSORT extensions for cluster randomised trials, non-inferiority and equivalence trials, non-pharmacological treatments, herbal interventions, and pragmatic trials. Additional extensions are forthcoming: for those and for up to date references relevant to this checklist, see www.consort-statement.org.

For peer review only - http://bmjopen.bmj.com/site/about/guidelines.xhtml

BMJ Open

Randomised feasibility trial into the effects of low frequency electrical muscle stimulation in advanced heart failure patients

Journal:	*BMJ Open*
Manuscript ID	bmjopen-2017-016148.R2
Article Type:	Research
Date Submitted by the Author:	05-Jul-2017
Complete List of Authors:	Ennis, Stuart; University Hospitals Coventry and Warwickshire NHS Trust, Cardiac Rehabilitation; Cardiff Metropolitan University, Cardiff Physiology and Health<br>McGregor, Gordon; University Hospitals Coventry and Warwickshire NHS Trust, Cardiac Rehabilitation; Coventry University Faculty of Health and Life Sciences<br>Hamborg, Thomas; University of Warwick Warwick Medical School,<br>Jones, Helen; Liverpool John Moores University, sport and exercise sciences<br>Shave, Rob; Cardiff Metropolitan University, Physiology and Health<br>Singh, Sally; University Hospitals of Leicester NHS Trust, Cardiac/Pulmonary Rehabilitation<br>Banerjee, Prithwish; University Hospitals Coventry and Warwickshire NHS Trust, Department of Cardiology; Coventry University
**Primary Subject Heading**:	Sports and exercise medicine
Secondary Subject Heading:	Cardiovascular medicine, Rehabilitation medicine
Keywords:	advanced heart failure, electrical muscle stimulation, neuromuscular electrical stimulation, cardiac rehabilitation, feasibility
Note: The following files were submitted by the author for peer review, but cannot be converted to PDF. You must view these files (e.g. movies) online.	
MAGIC PANTS Protocol V3 30 JUN 2014BMJ open.docm	

SCHOLARONE™
Manuscripts

Randomised feasibility trial into the effects of low frequency electrical muscle stimulation in advanced heart failure patients

Stuart Ennis[1,4], Gordon McGregor[1,6], Thomas Hamborg [2], Helen Jones[3], Robert Shave[4], Sally J Singh[5,6] and Prithwish Banerjee[1,6].

For peer review only

For peer review only - http://bmjopen.bmj.com/site/about/guidelines.xhtml

Abstract .

Objectives: Low Frequency Electrical Muscle Stimulation (LF-EMS) may have the potential to reduce breathlessness and increase exercise capacity in the chronic heart failure population who struggle to adhere to conventional exercise. The study's aim was to establish if a randomised controlled trial of LF-EMS was feasible.

Design and setting: Double blind (participants, outcome assessors), randomised study in a secondary care outpatient cardiac rehabilitation program.

Participants: Severe heart failure patients (New York Heart Association class III-IV) with left ventricular ejection fraction <40% documented by echocardiography were eligible.

Interventions: Participants were randomized (remotely by computer) to 8 weeks (5 x 60 mins per week) of either LF-EMS intervention (4Hz, continuous, n=30) or SHAM placebo (skin level stimulation only, n=30) of the quadriceps and hamstrings muscles. Participants used the LF-EMS straps at home and were supervised weekly

Outcome measures: Recruitment, adherence and tolerability to the intervention were measured during the trial as well as physiological outcomes (primary outcome: 6 minute walk, secondary outcomes: quadriceps strength, quality of life and physical activity).

Results: Sixty of 171 eligible participants (35.08%) were recruited to the trial. 12 (20%) of the 60 patients (4 LF-EMS, 8 SHAM) withdrew. Forty one patients (68.3 %), adhered to the protocol for at least 70% of the sessions**.** The physiological measures indicated no significant differences between groups in 6 minute walk distance,(P=0.13) and quality of life, (P=0.55) although both outcomes improved more with LF-EMS.

Conclusion: Severe heart failure patients can be recruited to and tolerate LF-EMS studies. A larger Randomised Controlled Trial (RCT) in the advanced heart failure population is technically feasible, although adherence to follow-up would be challenging. The preliminary improvements in exercise capacity and quality of life were minimal and this should be considered if planning a larger trial.

Trial registration number: ISRCTN16749049

Strengths and Limitations

1. To our knowledge, this was the first study to evaluate the design of a study into LF-EMS in advanced (NYHA III-IV) heart failure patients
2. Analysis of recruitment, retention and adherence in this hard to reach group contributes useful knowledge to the heart failure literature on how practical exercise interventions could be implemented.
3. This study was a real-world feasibility study. Advanced heart failure patients were recruited when deemed eligible by experienced clinicians based on available information. This approach can be subjective and lead to variability in disease severity in our sample. However this is in keeping with the pragmatic aim of our trial and provides external validity to our findings.
4. This study had a small sample size, and was not powered or designed to assess the effects of LF-EMS in advanced heart failure. The findings should therefore be considered preliminary.

For peer review only - http://bmjopen.bmj.com/site/about/guidelines.xhtml

Introduction

Chronic Heart Failure (CHF) affects approximately 26 million people worldwide, [1] and is associated with a poor prognosis; 30- 40% of patients diagnosed with heart failure die within a year. [2] Patients in New York Heart Association (NYHA) class III/IV are unable to perform the simplest daily activities, become depressed and have a poor quality of life.[3]

Regular aerobic exercise reduces breathlessness and muscle dysfunction for individuals with CHF whilst improving exercise capacity.[4,5,6] According to the ExTraMATCH meta-analysis,[7] exercise training leads to a 35% relative reduction in mortality, similar to the effects of beta-blockers[8] and ACE inhibitors.[9] However, those with advanced CHF are often so limited that they are unable to gain the holistic benefits of exercise.[4,7]

Electrical Muscle Stimulation (EMS) may provide an alternative rehabilitative therapy for this group. In patients with mild to moderate CHF, EMS can improve muscle strength of the legs, exercise capacity and quality of life. [10,11,12] Low frequency (4-5Hz) electrical muscle stimulation (LF-EMS) produces shivering-like sub-tetanic muscle contractions that can stimulate an aerobic response equivalent to 51% of maximal oxygen uptake.[13] Therapeutic levels of aerobic exercise can thus be achieved passively by LF-EMS,[14] and it has been shown to be comfortable and well tolerated in healthy individuals and those with mild to moderate CHF. [15,16] However, the impact of LF-EMS in advanced heart failure (NYHA class III/IV) patients is currently unknown. As advanced heart failure patients have shown poor uptake and adherence to intervention studies,[17] a preliminary study was needed to determine the feasibility of LF-EMS in this patient cohort prior to the development of a large-scale definitive trial.

Based upon recommendations for good practice in the design of pilot and feasibility studies [18] this study was undertaken with the following aims: To (a) test the robustness of the study protocol for a potential future trial, (b) estimate rates of recruitment, consent and retention, (c) determine the tolerability of the LF-EMS intervention and the effectiveness of the sham placebo in the NYHA III/IV CHF population, and (d) gain initial estimates of the efficacy of LF-EMS for all potential primary outcomes. This can be used for sample size calculations in future substantive trials.

For peer review only

Methods

Experimental Design

This feasibility study used a double blind parallel group randomised control design. Participants were randomised to either LF-EMS or 'sham' placebo for a period of eight weeks and blinded to group allocation. Outcomes were assessed at baseline (pre randomisation), eight weeks and 20 weeks follow-up.

Recruitment and screening

Between October 2013 and March 2015, University Hospital Coventry and Warwickshire, (UHCW) Hospital NHS Trust heart failure clinics lists were screened for patients fulfilling the eligibility criteria for the study. Sixty eligible participants were recruited. The study conformed to the Declaration of Helsinki and was approved by the local NHS Ethics Committee. All participants provided written informed consent.

Randomisation

The trial statistician, in conjunction with Warwick Clinical Trials Unit generated the randomisation sequence remotely (by computer) using permuted block randomisation. Group allocation was concealed from outcomes assessors and participants.

Participants

Male and female adults, >18 years old, with stable CHF, documented by echocardiography of left ventricular systolic dysfunction (ejection fraction < 40%) were eligible for the study. All participants had New York Heart Association (NYHA) functional class III-IV symptoms as judged by an experienced heart failure cardiologist. Participants were required to be medically stable, defined as the absence of hospital admission or alterations in medical therapy within the preceding two weeks. Exclusion criteria for safety and practical reasons were: (1) presence of implantable cardiac devices, (2) serious cardiac arrhythmias,(3) neurological disorders or previous stroke significant enough to limit exercise, (4) orthopaedic problems that prevented walking, (5) neuromuscular disease, (6) dementia or (7) a mid-thigh circumference of more than 50cm (due to the size of the LF-EMS straps).

LF-EMS Stimulation

The LF-EMS equipment (Biomedical Research Limited, Galway, Ireland) consisted of a pair of neoprene straps containing built-in adhesive gel electrodes. The equipment is CE marked under the European Medical Device Directive. The stimulator current waveform was designed to produce rhythmical contractions in the leg muscle groups occurring at a pulse frequency of 4-5Hz (pulse width: 620μs). The maximum peak output pulse current used was 140mA.

LF-EMS intervention

Participants used the LF-EMS or sham placebo for one hour, five times a week, for eight consecutive weeks. Of the five hourly sessions per week, four were

For peer review only - http://bmjopen.bmj.com/site/about/guidelines.xhtml

completed unsupervised in the participant's own home. The remaining session was conducted in a cardiac rehabilitation outpatient setting under the supervision of an exercise physiologist. The LF-EMS technology was retrospectively interrogated (i.e. at the weekly supervised sessions) to report date, frequency, duration and stimulation intensity.

'Sham' Placebo intervention

In the sham arm of the study, participants were provided with identical straps and electrodes. In contrast to the LF-EMS group the controller was programmed to deliver a very low level of stimulation (Frequency: 99Hz, pulse width: 150μs, maximum current amplitude: 7.3mA). This provided sensory input to the skin surface but little or no muscle activation. Participants in the sham group had the same induction, supervision and follow-up as the intervention arm.

Outcome Measures

Feasibility criteria

In relation to the design of pilot and feasibility studies, Thabane et al,[19] recommends stipulating criteria for success '*a priori*'. The feasibility criteria were:

1. *Recruitment rate* – At least 40% of eligible participants recruited to the trial

2. *Retention* – no more than 33% of participants drop out during the intervention period.

3. *Adherence* – 66% of participants tolerate the intervention and adhere to the protocol for ≥70% of the intervention period.

4. *Placebo efficacy*- Participants would be able to guess their group allocation no more often than would be expected by chance.

Primary outcome

Six Minute Walk Test (6MWT).

The 6MWT was conducted in accordance with the American Thoracic Society (ATS) guidelines.[20] Participants were instructed to walk as far as possible in six minutes along a 30m, flat, obstacle free corridor, turning 180 degrees at the end of every 30m. Standardised instructions and verbal encouragement were given.

Secondary outcomes

Isometric muscle strength

A hand held dynamometer (MicroFET2 Torque/Force indicator, Hoggan Health Industries, Utah, US) validated for assessing functional leg strength in elderly populations was used.[21] Participants sat in an elevated chair and were instructed to

For peer review only

maximally extend the knee while the assessor provided an equal and opposite resistive force, against the lower shin. Mean force generated was measured in Newtons.

Quality of Life: Minnesota Living with Heart Failure Questionnaire (MLHFQ)

The MLWHF questionnaire is a disease validated questionnaire,[22] that has been extensively used in heart failure studies. Questionnaire scores range from 0 to 105, with higher scores reflecting lower Quality of life. Participants were asked to answer each question based on their perception of health in the week previous to testing.

Physical Activity levels

Physical activity levels were measured by the Bodymedia© SenseWear Pro3 Armband. The multi-plane accelerometer was worn continuously for the seven days prior to testing to determine Total Energy Expenditure (TEE) per 24hr period was used as the main indicator of physical activity.

LF-EMS acceptability questionnaire

At the end of the trial participants were given a brief questionnaire used in previous LF-EMS studies,[13,14] to collect feedback on the acceptability of using LF-EMS regularly. Questions used the likert scale to discern cognitive and affective components of attitudes[23] about ease of use, comfort, tolerability and overall satisfaction.

Safety: Blood test

Venous blood samples were taken at baseline, four weeks and eight weeks to assess creatine kinase (CK), urea, and electrolytes. Participants would discontinue the trial if levels exceeded the upper limit of normal reference ranges

Data analysis

Data analyses for the feasibility objectives of this study were descriptive, based on the pre-determined levels specified above. Confidence intervals (set at 95%) were calculated for all secondary outcome measures in both groups and paired two-sample t-test conducted for between group comparisons. Intent-to-treat (ITT) analysis was employed in this study as is recommended for clinical trials.[24]

For peer review only - http://bmjopen.bmj.com/site/about/guidelines.xhtml

Results

Feasibility criteria outcomes

Recruitment

There were 171 eligible participants identified in the Coventry and Warwickshire area from November 2013 - April 2015. Sixty of 171 eligible participants (35.08%) were recruited to the trial. Participants were randomised and started on the trial during this period and were followed up until data collection finished in August 2015. Participant characteristics are presented in Table 1.

For peer review only

For peer review only - http://bmjopen.bmj.com/site/about/guidelines.xhtml

Table 1. Baseline demographic and clinical characteristics of the LF-EMS and sham placebo groups. Data presented as mean ± SD or absolute number and percent.

Demographics	LF-EMS (n-30)	Sham (n=30)
n Male	20 (66%)	22 (73%)
Age (yrs)	66.5 ± 7.8	66.8 ± 13.5
Body Mass Index (kg/m^2)	30.1 ± 4.9	27.8 ± 4.8
**Comorbidities**		
Prev MI/PCI/CABG	17 (56%)	11 (36%)
Diabetes	12 (40%)	10 (33%)
COPD	9 (30%)	8 (26%)
AF	20 (66%)	16 (53%)
Hypertension	13 (43%)	10 (33%
CKD	5 (16%)	13 (43%)
**Clinical**		
NT-pro-BNP (pg/mL)	3086 ± 3746	2046 ± 2545
Creatinine (μmol/L)	108 ± 49	113 ± 39
LVEF %	39 ± 11*	22 ± 12**
BP_{sys} (mmHg)	118 ± 16	126 ± 17
BP_{dia} (mmHg)	69 ± 9	74 ± 14
NYHA III	24 (80%)	22 (73%)
NYHA IV	6 (20%)	8 (26%)

NT-pro-BNP (pg/mL),N-terminal pro B-type natriuretic peptide; LVEF; left ventricular ejection fraction; BP_{sys} (mmHg), systolic blood pressure; BP_{dia} (mmHg), diastolic blood pressure; NYHA, New York Heart association; MI, myocardial infarction; PCI, percutaneous coronary intervention; CABG, coronary artery bypass graft surgery; COPD, chronic obstructive pulmonary disease; AF, atrial fibrillation; CKD, chronic kidney disease;

*n=10.Ejection fraction could not be accurately assessed in all patients due to poor body habitus/atrial fibrillation. An experienced cardiac sonographer made an ‘eyeball’ assessment of poor left ventricular function for all other participants

**n=5. See previous comments.

For peer review only

For peer review only - http://bmjopen.bmj.com/site/about/guidelines.xhtml

Retention

Twelve of the 60 participants (4 LF-EMS, 8 sham) (20%) withdrew and did not finish the intervention period (See Fig 1). Of these, only three found the intervention intolerable (1 LF-EMS, 2 sham). Other reasons for dropout were: deterioration in health (n= 6) family problems (n=2) and implantation of a cardioverter defibrillator (ICD) (n=1). Only 22 (45%) of those completing the intervention period returned for follow-up testing at 20 weeks. Reasons for non-follow-up were: deterioration in health (n=9), excluded due to implantation of cardiac resynchronisation therapy device (n=2), declined to take part without further explanation (n=13), and could not be contacted after repeated attempts (n= 3).

For peer review only

For peer review only - http://bmjopen.bmj.com/site/about/guidelines.xhtml

For peer review only

Figure 1. Flow diagram of a single centre blinded parallel group randomised feasibility trial of electrical muscle stimulation versus sham placebo in severe heart failure patients.

For peer review only - http://bmjopen.bmj.com/site/about/guidelines.xhtml

Adherence

Forty one (85.4 %) of the 48 participants (22-LF-EMS, 19-SHAM) who completed the intervention period (68.3% of the total sample) adhered to the strict protocol for the majority (>70%) of the eight weeks. Interrogation of the LF-EMS controllers revealed that participants in the LF-EMS group became more tolerant to the intervention; mean stimulation intensity increased from 57.79mA (95%CI: 51.16 to 64.42) during week 1 of the study to 84.86mA (95%CI: 75.44 to 94.28) by week 8, an improvement of 46.5%.

'Sham' Placebo

The sham placebo for the study appeared to be convincing as only 61% of participants guessed their treatment group correctly. The 95% confidence interval for the proportion of participants guessing correctly was (46% to 74%) and thus not significantly different from 50% which would be expected by chance. Furthermore, participants demonstrated an inclination to guess that they were randomised to LF-EMS regardless of group allocation.

Safety

No abnormalities were detected in CK, urea or electrolytes taken before, during or after the study. Likewise, no adverse events due to the intervention were recorded in either group.

Primary outcome- 6-minute walk test

Non-significant improvements after LF-EMS (8 week time point) and sham groups were observed in 6 MWD with a mean increase from baseline of 24m (P=0.13)in the LF-EMS group (Table 2.)

Secondary outcomes

Table 2 shows the mean values of the secondary outcome measures at each time point. There were no significant differences between groups in the change from baseline for any of the secondary outcome variables (Table 3). There was a non-significant improvement in quality of life in both groups.

For peer review only - http://bmjopen.bmj.com/site/about/guidelines.xhtml

For peer review only

314 Table 2: Outcome measurements – Time point averages and 95% confidence
315 intervals (CI)

Outcome	Time point	LF-EMS	Sham
Mean 6 MWD (metres) [95% CI]	Baseline	283 [237 – 328]	290 [243 – 337]
	(n)	29	29
	8 weeks	312 [262 – 362]	318 [270 – 365]
	(n)	26	22
	20 weeks	257 [173 – 342]	226 [126 – 325]
	(n)	12	10
(Mean leg strength (newtons) [95% CI]	Baseline	234.3 [196.5 – 272.]	297.5 [253 – 342]
	(n)	29	29
	8 weeks	224.9 [187.5 – 262.3]	321 [267.8 – 374.3]
	(n)	25	22
	20 weeks	181.6 [131.7 – 231.5]	207.1 [148.6 – 265.7]
	(n)	11	10
Mean QoL (score) [95% CI]	Baseline	53.1 [42.7 – 63.5]	50 [40 – 60.1]
	(n)	28	29
	8 weeks	43.9 [34.2 – 53.5]	43.1 [30.9 – 55.3]
	(n)	25	22
	20 weeks	51.7 [31.6 – 71.8]	37.0 [16.9 – 57]
	(n)	12	10
Mean TEE (joules) *[95% CI]*	Baseline	63,438 [56,170 – 70,705]	65,371 [59675 – 71,067]
	(n)	25	27
	8 weeks	59,783 [51,094 – 68,471]	59,687 [50,630 – 68,745]
	(n)	19	17
	20 weeks	61,878 [53,345 – 70,410]	63,541 [55,795 – 71,287]
	(n)	7	6

316 6 MWD, 6 minute walk distance, QoL, quality of life; TEE, Total Energy Expenditure

317

For peer review only - http://bmjopen.bmj.com/site/about/guidelines.xhtml

For peer review only

Table 3: -Changes from baseline averages and 95% confidence intervals (CI)

Outcome	Time point	LF-EMS	Sham	p-value
Mean 6 MWD (metres) [95% CI]	Baseline to 8 weeks	24 [9 – 40]	9 [-4 – 22]	0.1366
	(n)	26	22	
	Baseline to 20 weeks	0 [-32 – 31]	-26.30 [-63 – 11]	0.2409
	(n)	12	10	
(Mean leg strength (newtons) [95% CI]	Baseline to 8 weeks	-9.2 [-28.9 – 10.5]	6.0 [-19.3 – 31.4]	0.3244
	(n)	25	22	
	Baseline to 20 weeks	-43.4 [-78.7 – -8.2]	-74.1 [-116.3 – -31.9]	0.2223
	(n)	11	10	
Mean QoL (score) [95% CI]	Baseline to 8 weeks	-7.6 [-15.5 – 0.3]	-4.7 [-10.5 – 1.0]	0.5505
	(n)	25	22	
	Baseline to 20 weeks	1.5 [-12.5 – 15.7]	-14.0 [-34 – 6]	0.1610
	(n)	12	10	
Mean TEE (joules) [95% CI]	Baseline to 8 weeks	-4635 [-3963 – 4692]	-8168 [-14,342 – -1995]	0.5108
	(n)	19	17	
	Baseline to 20 weeks	1686 [-6435 – 9809]	4177 [-7695 – 16,050]	0.6634
	(n)	7	6	

6 MWD, 6 minute walk distance; QoL, quality of life; TEE, Total Energy Expenditure

For peer review only

Acceptability questionairre

Participants responses to the LF-EMS acceptability questionairre are summarised in Table 4. The mean response to putting on the straps was 2 ('quite easy') and the overall mean satisfaction of participants with the intervention was 6 out of 10. Mean responses to comfort, sensation, tolerabilty and continued use of LF-EMS were between 3 (medium) and 4 (quite hard/unpleasant).

Table 4. Mean responses to acceptability questionairre and standard deviations

Question	Mean response	
1. I found putting on the straps (1-easy, 5-hard)	2.0	(±1.17)
2. At the highest intensity I found the comfort level (1-acceptable, 5-unacceptable)	3.5	(±1.19)
3. Overall I found the sensation (1-pleasant, 5-unpleasant)	3.3	(±1.13)
4. I found putting on the LF-EMS for an hour (1-easy, 5-hard)	3.1	(±1.08)
5. I think I would find staying on a LF-EMS training routine (1-easy, 5-hard)	3.4	(±1.29)
6. Overall satisfaction with LF-EMS as a way of improving your fitness (1-none,10 extremely satisfied)	6.0	(±1.94)

Sample size calculation

The point estimate from the study and the upper CI limit of this estimate were calculated. The upper CI limit was used for the sample size calculation. For detecting the observed difference of 13.4 metres in this study a sample size of 240 patients per group would be required. However, a recent study [25] suggested that the minimal clinically important difference for 6MWD is 36 metres in mild-moderate CHF patients. The clinical benefit of the effect size in this study should be considered before proceeding with a larger trial

For peer review only

Discussion

The predetermined criteria for proceeding to a larger trial were achieved for dropout (20%), adherence (68.3%) and sham placebo efficacy (61.53% participants guessed correctly). However, only 35.06% of eligible patients were recruited, below the target of 40%. Initial outcome measures revealed no significant difference between intervention and placebo groups, although there was a non-significant improvement in 6MWD and quality of life after LF-EMS.

Feasibility outcomes

Recruitment

Percentage uptake (35.06%) of eligible patients in the study was below the predetermined criteria of 40%. This is similar to the poor uptake of conventional cardiac rehabilitation (CR) nationally in the UK: less than 40% of eligible heart failure patients accessed CR in the most recent National Audit of Cardiac Rehabilitation.[26]

Retention/adherence/tolerance

One strength of this study is the good level of adherence (68.3%) and retention (80%) compared with other clinical studies; In the HF-ACTION trial,[27] only 40% of patients in the exercise group (n=1159) reported adherence to recommended training volumes after three months. This may have been because of the ease of independent use at home of LF-EMS, in combination with the weekly supervised sessions with an exercise physiologist. The patients recruited in the present trial were more debilitated yet they engaged more with LF-EMS than those in the HF-ACTION trial, [27] suggesting that LF-EMS maybe more acceptable to this population than conventional exercise.

The dropout at 3 months follow-up was lower than expected due to ill health, device implantation and apathy, and would be challenging to overcome in a larger trial. Strategies to combat dropout could include combining assessment with clinical patient appointments to ensure compliance or arranging home visits for some assessments.

Feedback from the acceptability questionnaires may also be useful in curtailing dropout in a larger trial: the LF-EMS group generally thought that wearing the straps for an hour was ‘medium’ to ‘quite hard/unpleasant’. Continued use of a LF-EMS was deemed challenging also so it is possible that a reduced frequency of LF-EMS whilst still maintaining a sufficient dose e.g. 3 x 1 hr a week may enhance long term adherence.

Tolerance to the LF-EMS intervention improved during the study. Mean current intensity increased by 46% from week one to week eight. This tolerance effect is in keeping with an earlier study by Crognale, et al,[13] that showed a 20% increase in healthy active adults. The active adults tolerated higher absolute stimulation levels than in this study, both before and after habituation, suggesting that advanced CHF patients are subjectively less tolerant to LF-EMS than a healthy

For peer review only - http://bmjopen.bmj.com/site/about/guidelines.xhtml

population. In addition, the user feedback collected seems to support this view. Vivodtzev and colleagues,[28] examined factors determining tolerance of EMS in pulmonary patients. The study reported that lower tolerance to EMS was associated with greater severity of condition, fat free mass and inflammatory response. It is possible that the same is true in the CHF population but more research is needed to confirm this.

Outcome Measures

Baseline 6MWD was higher in our study sample than in other advanced heart failure studies.[29] This may have been due to high variability because of a few outliers in each group. This reflects the subjective nature of the NYHA classification system. However, signs and symptoms of advanced heart failure were primarily the eligibility criteria for this study and not 6MWD. In addition, the ≤300-m distance cutoff (below which our baseline mean falls) is often cited, as prognostically important and reflective of advanced disease in many investigations.[30,31,32] The non-significant improvements in exercise capacity as measured by 6 minute walk were smaller than those in a meta-analysis of EMS in heart failure patients by Smart, Dieberg and Gialluria.[10] These authors reported a combined improvement in 6MWD of 46.9m vs usual care or placebo, compared to the effect size of 13.2m in this study. However, patients in this study were more symptomatic than those included in the meta-analysis,[10] and thus had a lower baseline exercise capacity (286m vs 342m.) Nevertheless the mean relative increase (5%) in walk distance of participants in the LF-EMS group is within the measurement error associated with this test,[33] and probably should not be considered clinically significant.[25] The extrapolation from these results that severe CHF patients are beyond help from EMS maybe premature; a longer training period maybe required to show meaningful changes in exercise capacity, particularly as some participants took longer to tolerate meaningful EMS intensities than others.

Quality of life (MLHFQ) improved in both groups after the intervention. This may, in part, relate to the psychosocial benefits of engaging with researchers regularly in the cardiac rehabilitation facility.[34] The placebo effect of both interventions and its influence on patients' perception of well-being should not be underestimated.

Based on previous research by Banerjee et al,[15,16] and numerous high frequency EMS studies,[35,36,12] improvement in leg strength after use of LF-EMS was expected. The current trial however, showed no significant change in muscle strength. Muscle wasting, prevalent in many advanced heart failure patients,[37] could explain this observation. The chronic impairment of muscle tissue caused by heart failure affects the muscle and skin nerve receptors and hence contractility of the weakened muscle.[38] Participants with more functional leg muscles therefore, may have received greater stimulus to muscle tissue that others did for the same level of current intensity. This suggests that LF-EMS may not be effective for all advanced CHF patients.

Limitations

The sample for this study was small as is recommended for feasability studies[19] and this limits the external validity of our findings. Participants were deemed eligible for the study based on the judgment of experienced heart failure

For peer review only

clinicians using available knowledge. This may have led to greater variability in disease severity/limitation than was intended. The current amplitude (mA) stimulus intensity that participants chose to use was a limitation to the study design. Participants were instructed to adhere to the 'maximum tolerable intensity' during LF-EMS sessions. Due to considerable individual differences in the subjective perception of discomfort associated with EMS, It is therefore likely that there was variability in the intensity that individuals received

Conclusion

As some of the predetermined feasibility criteria were met in this trial, a larger study into the effects of LF-EMS on advanced heart failure patients could be undertaken. However this 'difficult to engage with' patient group would be very challenging to recruit and follow-up in sufficient numbers to provide definitive data on its efficacy. The improvements seen in this study in 6MWD, and quality of life measures, were not statistically significant. Leg strength and physical activity levels showed no significant change. A longer intervention period than 8 weeks could be considered, to give participants more time to adjust to the intervention. More investigation is required to determine which CHF patients are unresponsive to LF-EMS due to severe muscle dysfunction.

A larger trial may be feasible with this difficult population: however, it is unlikely that the non-significant improvement in exercise capacity and quality of life found in this pilot study justifies a larger pragmatic trial.

Acknowledgements

We would like to thank Matthew Annals for his recruitment expertise and for administering the intervention to participants. We would also like to thank Josie Goodby for her assistance and patience during all assessments.

Author affiliations

1(Department of Cardiac Rehabilitation) University Hospitals Coventry & Warwickshire NHS Trust, UK
2University of Warwick (Clinical Trials Unit), UK
3 Liverpool John Moores University, UK
4 Cardiff Metropolitan University, UK
5 University Hospitals of Leicester NHS trust, UK
6 University of Coventry, UK

Author contributions

SE, GM and PB contributed to the conception of the work. SE, GM, PB, SS, HJ, RS, and TH contributed to the design of the work. SE and GM contributed to the acquisition, of the work. SE, GM, PB, SS, HJ, RS, and TH contributed to the, analysis, or interpretation of data for the work. SE and GM drafted the manuscript. PB, SS, HJ, RS, and TH critically revised the manuscript. All gave final approval and agree to be accountable for all aspects of work ensuring integrity and accuracy

Funding

For peer review only - http://bmjopen.bmj.com/site/about/guidelines.xhtml

This study was funded by a National Institute for Health Research (NIHR) Research for Patient Benefit (RfPB) award.

Declaration of Conflicting Interests

The Author(s) declare(s) that there is no conflict of interest

Ethics approval NRES Committee West Midlands - Coventry & Warwickshire13/WM/0240

Disclaimer

This article/paper/report presents independent research funded by the National Institute for Health Research (NIHR). The views expressed are those of the author(s) and not necessarily those of the NHS, the NIHR or the Department of Health.

Data sharing statement

All available data can be obtained by contacting the corresponding author:

stuart.ennis@uhcw.nhs.uk

References

1. Ambrosy, AP, Fonarow, GC, Butler J et al. The global health and economic burden of hospitilizations for heart failure: lessons learned from HHF registries. *J Am Coll Cardiol.* 2014; 63:1123-33

2. NICE 2010 guideline:CG108 Chronic heart failure (2010). available at: http://guidance.nice.org.uk/CG108NICEGuidance/pdf/English (accessed January 2016)

3. Kop WJ, Synowski SJ, Gottlieb SS. Depression in heart failure: biobehavioral mechanisms. *Heart Fail Clin*. 2011;7(1): 23-38.

4. Taylor RS, Sagar VA, Davies EJ et al. Exercise-based rehabilitation for heart failure. *Cochrane Database Syst Rev*. 2014;(4):CD003331. doi: 10.1002/14651858.CD003331.pub4

5. Piepoli M, Conraads V, Corrà U et al. Exercise training in heart failure: from theory to practice. A consensus document of the Heart Failure Association and the

For peer review only - http://bmjopen.bmj.com/site/about/guidelines.xhtml

European Association for Cardiovascular Prevention and Rehabilitation. *Eur J Heart Fail*. 2011;13:347–357

6. Höllriegel R, Winzer EB, Linke A et al. Long-Term Exercise Training in Patients With Advanced Chronic Heart Failure: SUSTAINED BENEFITS ON LEFT VENTRICULAR PERFORMANCE AND EXERCISE CAPACITY *J Cardiopulm Rehabil Prev.* 2016;36(2):117-24

7. Piepoli MF, Davos C et al. ExTraMATCH Collaborative Exercise training meta-analysis of trials in patients with chronic heart failure (ExTraMATCH). *BMJ.* 2004;1(24): 328(7433):189

8. Sullach JA, Goldstein S. Use of beta-blockers in chronic heart failure. *Ann Med*. 2003; 35: 259-66.

9. Flather MD, Yusuf S, Køber L,et al. Long term ACE inhibitor therapy in patients with heart failure or left ventricular dysfunction: a systemic overview of the data from individual patients. ACE-Inhibitor Myocardial Infarction Collaborative Group *Lancet.* 2000;355:1578-81.

10. Smart NA, Dieberg G and Giallauria F. Functional electrical stimulation for chronic heart failure: a meta-analysis. *Int J Cardiol.* 2013;167(1): 80-86.

11. Nuhr, MJ, Pette D, Berger R et al. Beneficial effects of chronic low-frequency stimulation of thigh muscles in patients with advanced chronic heart failure. *Eur Heart J.* 2004;25(2): 136-143.

12. Dobsak P, Novakova M, Siegelova J et al. Low-frequency electrical stimulation increases muscle strength and improves blood supply in patients with chronic heart failure. *Circ J.* 2006;70(1): 75-82.

13. Crognale D, Vito GD, Grosset JF et al. (2013). Neuromuscular electrical stimulation can elicit aerobic exercise response without undue discomfort in healthy physically active adults. *J Strength Cond Res.* 2013; 27(1): 208-215.

For peer review only

For peer review only - http://bmjopen.bmj.com/site/about/guidelines.xhtml

14. Minogue CM, Caulfield BM, Lowery MM. Whole body oxygen uptake and evoked torque during sub-tetanic isometric electrical stimulation of the quadriceps muscles in a single 30-minute session. *Arch Phys Med Rehabil*. 2014; 95(9):1750-8.

15. Banerjee P, Clark A, Witte K et al. Electrical stimulation of unloaded muscles causes cardiovascular exercise by increasing oxygen demand. *Eur J Cardiovasc Prev Rehabil.* 2005;12(5): 503-508.

16. Banerjee P, Caulfield B, Crowe L et al. Prolonged electrical muscle stimulation exercise improves strength, peak VO_2, and exercise capacity in patients with stable chronic heart failure. *J Card Fail.* 2009;15(4): 319-326.

17. Nieminen MS, Dickstein K, Fonseca C et al. The patient perspective: Quality of life in advanced heart failure with frequent hospitalisations. *Int J Cardiol.* 2015; 191:256-64.

18. Lancaster GA, Dodd S, Williamson PR. Design and analysis of pilot studies: recommendations for good practice. *J Eval Clin Pract.* 2004;10(2):307-12.

19. Thabane L, Ma J, Chu R, et al. A tutorial on pilot studies: The what, why and How. *BMC Medical Research Methodology.* 2010; (2)10:1.

20. Holland AE, Spruit MA, Troosters T et al. An official European Respiratory Society/American Thoracic Society technical standard: field walking tests in chronic respiratory disease. *Eur Respir J.* 2014;44(6): 1428-46.

21. Schaubert KL and Bohannon RW. Reliability and validity of three strength measures obtained from community-dwelling elderly persons *J Strength Cond Res.* 2005;19(3): 717-720

22. Rector TS and Cohn JN. Assessment of patient outcome with the Minnesota Living with Heart Failure questionnaire: reliability and validity during a randomized, double-blind, placebo-controlled trial of pimobendan. Pimobendan Multicenter Research Group. *Am Heart J.* 1992;124(4): 1017-1025.

For peer review only - http://bmjopen.bmj.com/site/about/guidelines.xhtml

23. Likert, R. *A Technique for the Measurement of Attitudes*. New York: The Science Press, 1932.

24. Alshurafa M, Briel M, Akl EA et al. Inconsistent definitions for intention-to-treat in relation to missing outcome data: systematic review of the methods literature. *PLoS One.* 2012; 7(11): e49163.

25. Täger T, Hanholz W, Cebola R, et al. Minimal important difference for 6-minute walk test distances among patients with chronic heart failure. *Int J Cardiol*. 2014;176(1):94-8.

26. The National Audit of Cardiac Rehabilitation—Annual Statistical Report 2015-2016. Available from http://www.cardiacrehabilitation.org.uk/docs/2007.pdf,

27. O'Connor CM, Whellan DJ, Lee KL et al. Efficacy and safety of exercise training in patients with chronic heart failure: HF-ACTION randomized controlled trial. *JAMA.* 2009; 301(14): 1439-1450.

28. Vivodtzev I, Rivard B, Gagnon P et al. Tolerance and physiological correlates of neuromuscular electrical stimulation in COPD: a pilot study. *PLoS One.* 2014; 9;9(5):e94850. doi: 10.1371/journal.pone.0094850

29. Reeves GR, Whellan DJ, O'Connor CM et al. A Novel Rehabilitation Intervention for Older Patients With Acute Decompensated Heart Failure *JACC: Heart Failure* 2017, 613; DOI: 10.1016/j.jchf.2016.12.019

30. Guazzi M, Dickstein K, Vicenzi R and Arena R. Six-Minute Walk Test and Cardiopulmonary Exercise Testing in Patients With Chronic Heart Failure. *Circulation: Heart Failure*. 2009; 2:549-555.

31. Arslan S, Erol MK, Gundogdu F et al. Prognostic value of 6-minute walk test in stable outpatients with heart failure. *Tex Heart Inst J.* 2007; **34**: 166–169.

32. Rostagno C, Olivo G, Comeglio M, et al. Prognostic value of 6-minute walk corridor test in patients with mild to moderate heart failure: comparison with other methods of functional evaluation.2003 *Eur J Heart Fail.* **5**: 247–252.

For peer review only

For peer review only - http://bmjopen.bmj.com/site/about/guidelines.xhtml

33. Zugck C, Krüger C, Dürr S et al. Is the 6-minute walk test a reliable substitute for peak oxygen uptake in patients with dilated cardiomyopathy? *Eur Heart J*. 2000; 21(7):540-9

34. Jeon YH, Kraus SG, et al. The experience of living with chronic heart failure: a narrative review of qualitative studies. *BMC Health Serv Res*. 2010 24;10:77

35. Quittan M, Wiesinger GF, Sturm B et al. Improvement of thigh muscles by neuromuscular electrical stimulation in patients with refractory heart failure: a single-blind, randomized, controlled trial *Am J Phys Med Rehabil.* 2001;80(3): 206-214

36. Harris S, LeMaitre JP, Mackenzie G et al. A randomised study of home-based electrical stimulation of the legs and conventional bicycle exercise training for patients with chronic heart failure. *Eur Heart J.* 2003;24(9): 871-878.

37. Fülster S, Tacke M, Sandek A et al. Muscle wasting in patients with chronic heart failure: results from the studies investigating co-morbidities aggravating heart failure (SICA-HF). *Eur Heart J*. 2013;34(7): 512-9.

38. Rullman E, Andersson DC, Melin M et al. Modifications of skeletal muscle ryanodine receptor type 1 and exercise intolerance in heart failure. *J Heart Lung Transplant*. 2013;32(9):925-9. doi: 10.1016/j.healun.2013.06.026

For peer review only - http://bmjopen.bmj.com/site/about/guidelines.xhtml

For peer review only

For peer review only - http://bmjopen.bmj.com/site/about/guidelines.xhtml

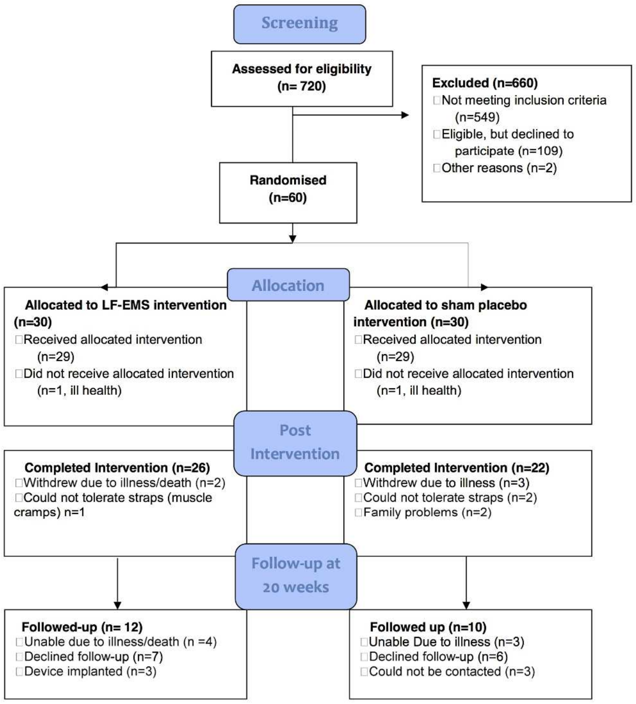

Figure 1. Flow diagram of a single centre blinded parallel group randomised feasibility trial of electrical muscle stimulation versus sham placebo in severe heart failure patients.

209x297mm (300 x 300 DPI)

CONSORT 2010 checklist of information to include when reporting a randomised trial*

Section/Topic	Item No	Checklist item	Reported on page No
**Title and abstract**			
	1a	Identification as a randomised trial in the title	1
	1b	Structured summary of trial design, methods, results, and conclusions (for specific guidance see CONSORT for abstracts)	2
**Introduction**			
Background and objectives	2a	Scientific background and explanation of rationale	3
	2b	Specific objectives or hypotheses	3
**Methods**			
Trial design	3a	Description of trial design (such as parallel, factorial) including allocation ratio	3
	3b	Important changes to methods after trial commencement (such as eligibility criteria), with reasons	n/a
Participants	4a	Eligibility criteria for participants	4
	4b	Settings and locations where the data were collected	4
Interventions	5	The interventions for each group with sufficient details to allow replication, including how and when they were actually administered	4,5
Outcomes	6a	Completely defined pre-specified primary and secondary outcome measures, including how and when they were assessed	5,6
	6b	Any changes to trial outcomes after the trial commenced, with reasons	n/a
Sample size	7a	How sample size was determined	
	7b	When applicable, explanation of any interim analyses and stopping guidelines	n/a
Randomisation:			
Sequence generation	8a	Method used to generate the random allocation sequence	4
	8b	Type of randomisation; details of any restriction (such as blocking and block size)	4
Allocation concealment mechanism	9	Mechanism used to implement the random allocation sequence (such as sequentially numbered containers), describing any steps taken to conceal the sequence until interventions were assigned	4
Implementation	10	Who generated the random allocation sequence, who enrolled participants, and who assigned participants to interventions	4
Blinding	11a	If done, who was blinded after assignment to interventions (for example, participants, care providers, those	4

For peer review only - http://bmjopen.bmj.com/site/about/guidelines.xhtml

		assessing outcomes) and how	
	11b	If relevant, description of the similarity of interventions	4
Statistical methods	12a	Statistical methods used to compare groups for primary and secondary outcomes	6
	12b	Methods for additional analyses, such as subgroup analyses and adjusted analyses	n/a
**Results**			
Participant flow (a diagram is strongly recommended)	13a	For each group, the numbers of participants who were randomly assigned, received intended treatment, and were analysed for the primary outcome	
	13b	For each group, losses and exclusions after randomisation, together with reasons	
Recruitment	14a	Dates defining the periods of recruitment and follow-up	7
	14b	Why the trial ended or was stopped	n/a
Baseline data	15	A table showing baseline demographic and clinical characteristics for each group	12
Numbers analysed	16	For each group, number of participants (denominator) included in each analysis and whether the analysis was by original assigned groups	10
Outcomes and estimation	17a	For each primary and secondary outcome, results for each group, and the estimated effect size and its precision (such as 95% confidence interval)	9,10,11,12
	17b	For binary outcomes, presentation of both absolute and relative effect sizes is recommended	n/a
Ancillary analyses	18	Results of any other analyses performed, including subgroup analyses and adjusted analyses, distinguishing pre-specified from exploratory	n/a
Harms	19	All important harms or unintended effects in each group (for specific guidance see CONSORT for harms)	11
**Discussion**			
Limitations	20	Trial limitations, addressing sources of potential bias, imprecision, and, if relevant, multiplicity of analyses	15
Generalisability	21	Generalisability (external validity, applicability) of the trial findings	14
Interpretation	22	Interpretation consistent with results, balancing benefits and harms, and considering other relevant evidence	14,15
**Other information**			
Registration	23	Registration number and name of trial registry	
Protocol	24	Where the full trial protocol can be accessed, if available	n/a
Funding	25	Sources of funding and other support (such as supply of drugs), role of funders	16

*We strongly recommend reading this statement in conjunction with the CONSORT 2010 Explanation and Elaboration for important clarifications on all the items. If relevant, we also recommend reading CONSORT extensions for cluster randomised trials, non-inferiority and equivalence trials, non-pharmacological treatments, herbal interventions, and pragmatic trials. Additional extensions are forthcoming: for those and for up to date references relevant to this checklist, see www.consort-statement.org.

For peer review only - http://bmjopen.bmj.com/site/about/guidelines.xhtml
